# Complex Landscape of Germline Variants in Brazilian Patients With Hereditary and Early Onset Breast Cancer

**DOI:** 10.3389/fgene.2018.00161

**Published:** 2018-05-07

**Authors:** Giovana T. Torrezan, Fernanda G. dos Santos R. de Almeida, Márcia C. P. Figueiredo, Bruna D. de Figueiredo Barros, Cláudia A. A. de Paula, Renan Valieris, Jorge E. S. de Souza, Rodrigo F. Ramalho, Felipe C. C. da Silva, Elisa N. Ferreira, Amanda F. de Nóbrega, Paula S. Felicio, Maria I. Achatz, Sandro J. de Souza, Edenir I. Palmero, Dirce M. Carraro

**Affiliations:** ^1^Laboratory of Genomics and Molecular Biology, International Research Center, CIPE/A.C. Camargo Cancer Center, São Paulo, Brazil; ^2^National Institute for Science and Technology in Oncogenomics and Therapeutic Innovation, São Paulo, Brazil; ^3^Laboratory of Bioinformatics and Computational Biology, International Research Center, CIPE/A.C. Camargo Cancer Center, São Paulo, Brazil; ^4^Instituto de Bioinformática e Biotecnologia−2bio, Natal, Brazil; ^5^Instituto Metrópole Digital, Federal University of Rio Grande do Norte, Natal, Brazil; ^6^Bioinformatics Multidisciplinary Environment, Federal University of Rio Grande do Norte, Natal, Brazil; ^7^Research and Development, Fleury Group, São Paulo, Brazil; ^8^Oncogenetics Department, A.C. Camargo Cancer Center, São Paulo, Brazil; ^9^Molecular Oncology Research Center, Barretos Cancer Hospital, São Paulo, Brazil; ^10^Clinical Genetics Branch, Division of Cancer Epidemiology and Genetics, National Cancer Institute, National Institutes of Health, Department of Health and Human Services, Bethesda, MD, United States; ^11^Brain Institute, Federal University of Rio Grande do Norte, Natal, Brazil; ^12^Barretos School of Health Sciences, Dr. Paulo Prata – FACISB, Barretos, Brazil

**Keywords:** cancer predisposition genes, hereditary breast cancer, whole-exome sequencing, germline pathogenic variants, cancer susceptibility, DNA repair genes

## Abstract

Pathogenic variants in known breast cancer (BC) predisposing genes explain only about 30% of Hereditary Breast Cancer (HBC) cases, whereas the underlying genetic factors for most families remain unknown. Here, we used whole-exome sequencing (WES) to identify genetic variants associated to HBC in 17 patients of Brazil with familial BC and negative for causal variants in major BC risk genes (*BRCA1/2, TP53*, and *CHEK2* c.1100delC). First, we searched for rare variants in 27 known HBC genes and identified two patients harboring truncating pathogenic variants in *ATM* and *BARD1*. For the remaining 15 negative patients, we found a substantial vast number of rare genetic variants. Thus, for selecting the most promising variants we used functional-based variant prioritization, followed by NGS validation, analysis in a control group, cosegregation analysis in one family and comparison with previous WES studies, shrinking our list to 23 novel BC candidate genes, which were evaluated in an independent cohort of 42 high-risk BC patients. Rare and possibly damaging variants were identified in 12 candidate genes in this cohort, including variants in DNA repair genes (*ERCC1* and *SXL4*) and other cancer-related genes (*NOTCH2, ERBB2, MST1R*, and *RAF1*). Overall, this is the first WES study applied for identifying novel genes associated to HBC in Brazilian patients, in which we provide a set of putative BC predisposing genes. We also underpin the value of using WES for assessing the complex landscape of HBC susceptibility, especially in less characterized populations.

## Introduction

Hereditary breast cancer (HBC) corresponds to ~5–10% of all breast cancer cases (Honrado et al., 2005). The most common breast cancer predisposing syndrome is hereditary breast and ovarian cancer syndrome (HBOC) that is related to pathogenic germline variants in *BRCA1* (OMIM 113705) and *BRCA2* (OMIM 600185) genes (Anglian Breast Cancer Study, 2000). These genes correspond to ~20–25% of all HBC (Anglian Breast Cancer Study, 2000; Kean, 2014; Silva et al., 2014). Besides *BRCA1/2* genes, pathogenic variants in other high- and moderate-risk genes, such as *TP53, CHEK2, ATM, STK11, PALB2*, among others, also lead to an increased breast cancer (BC) risk, revealing a high complexity in breast cancer predisposition (Elledge and Allred, 1998; Meijers-Heijboer et al., 2002; Walsh and King, 2007).

To date, over 35 genes have been suggested to carry high and/or moderate BC risk variants (OMIM, 2015^1^; Shiovitz and Korde, 2015). However, only a minority of these genes have an established significant association demonstrated by both stringent burden testing and statistical analyses (Easton et al., 2015). Moreover, despite extensive sequencing efforts, variants in known BC susceptibility genes are present in <30% of BC cases with positive family history or an early age of onset (Shiovitz and Korde, 2015; Chandler et al., 2016), meaning that the underlying genetic factors for most HBC remain unknown.

In the past few years, advances in next-generation sequencing (NGS), specially whole-exome sequencing (WES), have led to the identification of causative variants in several rare familial syndromes, including hereditary cancer (Comino-Méndez et al., 2011; Seguí et al., 2015). Up to the present time, more than 16 different WES studies (both family-based and case studies) have been carried out for HBC, and a few novel BC susceptibility genes were identified: *XRCC2, RINT1, RECQL*, and *FANCM* (Chandler et al., 2016). Nevertheless, the small number of novel major BC autosomal dominant predisposing genes disclosed in these studies has pointed to the possible existence of very rare, or even particular, high and moderate penetrant variants. Conversely, other forms of inheritance, such as recessive and oligogenic transmission of cancer predisposition, cannot be discarded (Sokolenko et al., 2015). In this sense, further WES investigation in different families or populations is crucial for expanding the catalog of breast tumor predisposing genes.

In two previous studies of our group, we screened young BC women (Carraro et al., 2013) and women with clinical criteria of HBOC (Silva et al., 2014) for pathogenic variants in the complete coding sequence of *BRCA1, BRCA2*, and *TP53* genes, and for *CHEK2* c.1100delC point mutation, detecting 22–26% of pathogenic variant carriers. Both studies disclosed a large number of women negative for pathogenic variants in the most important genes associated with BC risk, claiming for the necessity of identifying rare and/or novel BC predisposing genes. Thus, the aim of the current study was to investigate, by WES, breast cancer patients with clinical criteria for HBOC and without pathogenic variants in major breast cancer predisposing genes, using rigorous functional criteria for selection of detected variants, in order to identify the most promising new HBC-causing genes.

## Materials and methods

### Patients and controls

WES was performed in 17 patients from A.C. Camargo Cancer Center (15 unrelated patients and two siblings) diagnosed with BC and fulfilling one or more of the following criteria of HBOC syndrome: early onset BC (<36 years); bilateral BC; breast plus another primary related tumor (ovary, fallopian tube or primary peritoneal tumors). These patients were selected from previous studies (Carraro et al., 2013; Silva et al., 2014) from our group and were negative for pathogenic variants in *BRCA1/2, TP53*, and *CHEK2* c.1100delC. Two patients (including the two sisters) were carriers of variants of uncertain clinical significance (VUS) in *BRCA1* gene. The detailed inclusion criteria from both studies were described previously (Carraro et al., 2013; Silva et al., 2014). One affected woman of one family participated in the cosegregation study for specific candidate variants.

**Figure 1:**
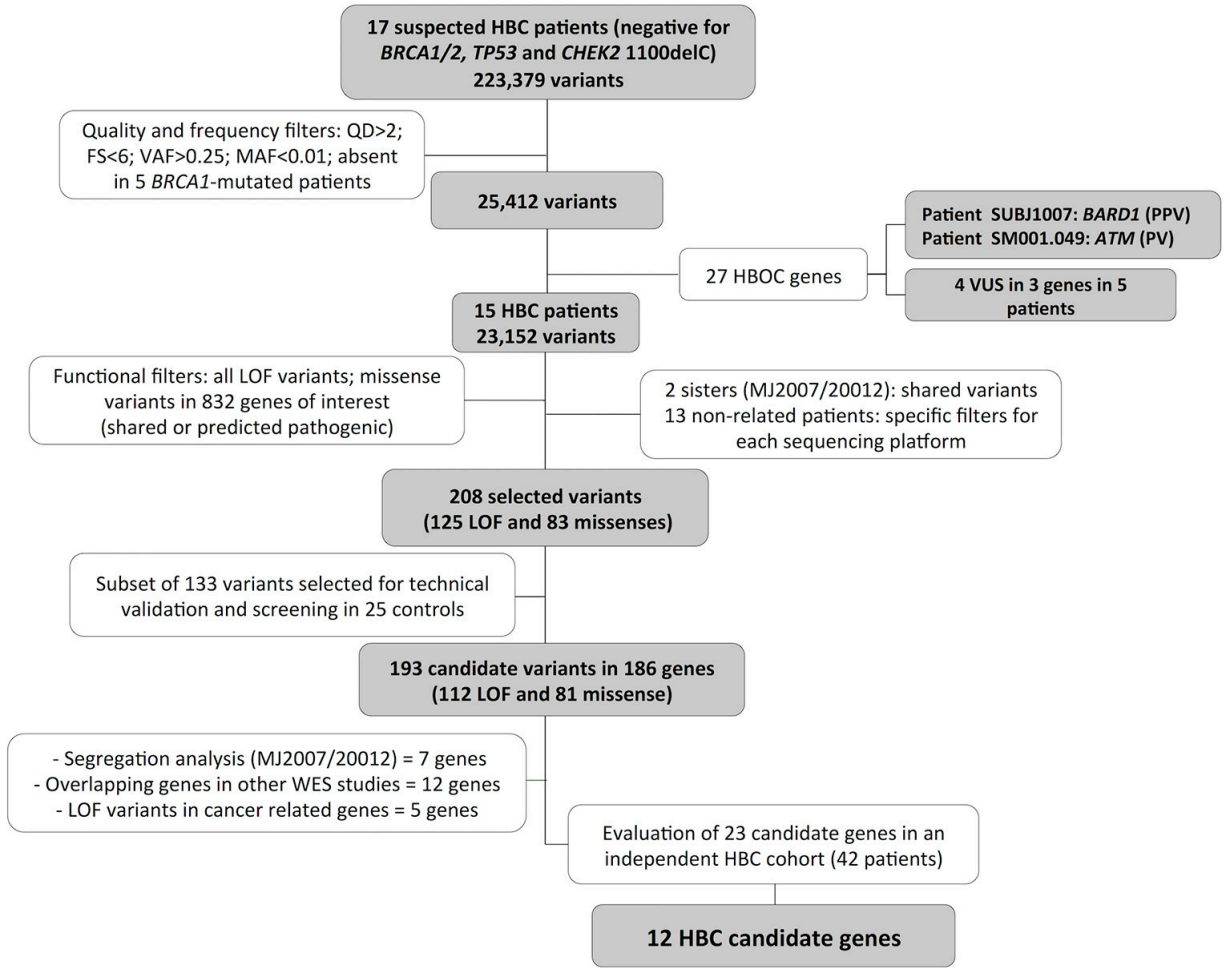
Variants selection workflow. WES data from 17 breast cancer patients were analyzed using quality, frequency, and functional based filters, resulting in 186 breast cancer predisposing candidate genes. A final 23 candidate genes were entirely investigated for LOF or possibly pathogenic variants in 42 additional BC patients suspected of HBC and negative for mutations in the major predisposing genes, resulting in 12 final HBC candidate genes. FS, FisherStrand; QD, QualByDepth; PPV, probably pathogenic variant; PV, pathogenic variant; HBOC, Hereditary Breast and Ovarian Cancer; LOF, loss of function; SA, segregation analysis; WES, whole exome sequencing.

Five germline *BRCA1*-mutation carriers that were submitted to WES in the same platform were included for variant filtering. For validation of selected variants, target NGS validation was applied in 25 healthy women without family history of cancer, considered here as a control group. Additionally, a selected number of candidate genes were screened in an independent group of 42 patients at risk for HBC from a distinct project, obtained from Barretos Cancer Hospital (Barretos, São Paulo, Brazil). Figure 1 depicts the study design and workflow, describing the projects steps and the analysis performed in each patients and controls groups.

All participants signed an informed consent. This study was performed in accordance with the Helsinki Declaration and was approved by the A.C. Camargo Cancer Center (1754/13) and Barretos Hospital (916/2015) ethics committees.

### DNA isolation

Genomic DNA was obtained from A.C. Camargo Cancer Center Biobank. In brief, DNA was extracted from peripheral leukocytes by Puregene®-DNA purification Kit (Qiagen, Hilden, Germany), according to manufacturer's instructions. DNA concentration, purity and integrity were assessed by spectrophotometry (Nanodrop 2000—Thermo Fisher Scientific, Waltham, MA) and fluorometry (Qubit—Life Technologies, Foster City, CA, USA).

### Whole exome sequencing

For the 17 patients of the discovery set, WES was performed using the SOLiD and/or Ion Proton platforms. For SOLiD exomes, libraries were prepared using SOLiD™ Fragment Library Barcoding Kit (Life Technologies) and SureSelect Human All Exon V4 Kit 50 Mb (Agilent Technologies), according to the manufacturer's instructions. Sequencing of paired-end libraries (50 X 75 bp) was performed in a Solid 5500XL System (Life Technologies). For Ion Proton exomes, libraries were prepared using Ion Xpress™ Plus Fragment Library Kit and Ion TargetSeq™ Exome Kit (Thermo Fisher Scientific), according to the manufacturer's instructions. Each Ion Proton exome library was sequenced on Ion Proton instrument using Ion PI Sequencing 200 Kit v3 and Ion PI Chip v3 (Thermo Fisher Scientific). The resulting sequences were mapped to the reference genome (GRCh37/hg19). Base Calling and alignment were performed by SOLiD™ BioScope 1.2™ Software (Life Technologies) (SOLID data) and by Torrent Suite v4.2 server (Ion Proton data). Variant calling and annotation were done by GATK (Genome Analysis Toolkit) pipeline made available by the Broad Institute. The data obtained in this study is available at Sequence Read Archive (SRP120031).

### Variants selection and prioritization

For variant filtering, identified variants were annotated with VarSeq (Golden Helix) against reference databases (RefSeq, 1000Genomes, ESP6500, ExAC, dbSNP, and ClinVar). First, for quality filtering, we selected variants with QD > 2 (QD = variant call confidence normalized by depth of sample reads supporting a variant), FS < 6 (FS = strand bias estimated by GATK using Fisher's Exact Test), base coverage ≥ 10x, variant allele frequency (VAF) > 0.25. For four patients with data from both Solid and Ion Proton, only variants detected in both platforms were selected. For one patient with data exclusively from Ion Proton, variants occurring in regions of homopolymer > 4 bases were excluded. Qualified variants were excluded if present in five *BRCA1*-mutation carriers patients analyzed by WES in Solid 5500, and variants present in population databases with frequency > 1% (minor allele frequency [MAF] > 0.01), as well as variants present in more than three unrelated patients. Finally, a recently public available Brazilian database of WES from 609 healthy individuals (Abraom—Brazilian genomic variants; http://abraom.ib.usp.br/) was also used for manually excluding population-specific variants (MAF > 0.01).

Next, for a function-based prioritization, we selected variants leading to loss of function in any gene (frameshift indels, stop codon, and canonical splice site variants) and missense or in-frame indels variants in 832 genes of interest. These genes were selected from commercial panels targeting somatic and germline cancer mutated genes, consensus cancer genes previously described (Futreal et al., 2004) and genes from DNA repair pathways (from KEGG and Putnam et al., 2016) (Supplementary Table 1). For the two related patients, any shared missense or in-frame indels variants in these 832 genes were selected. For the 15 unrelated patients, we selected only variants predicted to be damaging in at least four out of six variant effect prediction software. For these analyses, the results from the following tools were obtained using VarSeq: SIFT, Polyphen v2, Functional Analysis through Hidden Markov Models (FATHAMM and FATHAMM-MKL), MutationAssessor and MutationTaster. Additionally, we analyzed the potential effect on splicing of the selected LOF and missense variants using dbscSNV annotations (cut-off > 0.6 in ADA and/or RF scores).

### Sanger validation

Two pathogenic variants (PV) or probably pathogenic variants (PPV) in *BARD1* and *ATM* were validated by Sanger sequencing. Briefly, 50 ng of leukocyte DNA was submitted to PCR performed with GoTaq Green Master Mix (Promega), cleaned with ExoSAP-IT (USB Corporation) and sequenced in both directions with BigDye Terminator v3.1 (Life Technologies) using an ABI 3130xl DNA sequencer (Life Technologies), according to manufacturer's instructions. The sequencing results were aligned using CLCBio Genomics Workbench Software (CLCBio, Qiagen). Primer sequences are available under request.

### Targeted NGS validation

A subset of 139 variants (Supplementary Table 2) selected from exome data were validated by multiplex targeted NGS using a custom Ion AmpliSeq panel. Primers were designed using Ion AmpliSeq Designer v3.0.1 (Life Technologies). Libraries were prepared with 20 ng of DNA from each patient using Ion AmpliSeq™ Library Kit 2.0 (Life Technologies). Sequencing was performed using either Ion PGM or Ion Proton platforms, according to the manufacturer's instructions. Sequencing reads mapped to the human genome reference (hg19) using Torrent Suite Browser 4.0.1. On average 166,697 mapped reads were obtained per sample, yielding a mean targeted base coverage of 156X (ranging from 54 to 450). Variants were identified using the VariantCaller v4.0.r73742 plugin and confirmed using CLC Genomics Workbench software (Qiagen). The identified variants were considered if base coverage was ≥10x and VAF > 25%.

To filter out genetic variants common in Brazilian population, the validated variants were evaluated in control group of 25 healthy women by using the same panel. For that, pools of five equimolar genomic DNA samples were prepared by containing 4 ng of each patient (five patients per pool). Libraries preparation, sequencing and mapping were performed as described above. On average 928,194 mapped reads were obtained per pool (mean targeted base coverage 1114X; ranging from 990 to 1,314). Variant calls were obtained using the VariantCaller v4.0.r73742 plugin applying the following filter parameters: VAF > 2%; variant coverage ≥10X.

### Cosegregation analysis

For one family in which a segregation analysis was feasible, DNA from one additional affected individual was obtained. The cosegregation study of specific variants was performed using the same custom gene panel and protocol described previously or with amplicon based library construction and sequencing in Ion Proton platform.

### Independent cohort validation

For screening the HBC predisposing candidate genes selected in this study an independent cohort comprised of 42 breast cancer patients at risk for HBC from Barretos Cancer Hospital was used. These samples were analyzed through WES in a parallel study using Nextera Rapid Capture Expanded Exome and NextSeq 500 System (Illumina, San Diego, CA). In these data, we assessed the entire coding regions of the 23 genes disclosed in this study for the presence of rare and possibly pathogenic variants, using the same criteria as in our discovery cohort.

## Results

In this study we used WES to disclose variants contributing to BC increased risk in patients fulfilling stringent clinical criteria indicating a genetic predisposition to BC and that were negative for pathogenic variants in four major BC genes (*BRCA1/2, TP53*, and *CHEK2* 1100delC). The clinical features and family history of cancer for the 17 selected patients are described in Supplementary Table 3.

For the WES, an average of 46,307,427 sequence reads was obtained for each patient and 75.7% (average) of the target bases were covered by 10 or more reads (Supplementary Table 4). More than 200,000 variants were identified in these patients. To prioritize the identified variants, we applied several filters focusing on quality, frequency and function of the identified alterations. The workflow of the variant prioritization is depicted in Figure 1 and the details of used filters are described in the Materials and Methods section.

Regarding frequency filters, we excluded variants with a minor allele frequency (MAF) >1% in public databases or those present in five germline *BRCA1*-mutation carriers sequenced in our facility, assuming that these variants represent benign or low-penetrance variants. Following these initial data filtering, 25,412 were identified.

### Variants in moderated and high penetrance breast cancer genes

Initially, we used WES data to search for rare variants in 27 well-established and emerging HBC predisposing genes (the four previously evaluated genes (*BRCA1/2, TP53*, and *CHEK2* c.1100delC) and 23 additional genes): *ATM, BARD1, BLM, BRCA1, BRCA2, BRIP1, CDH1, CHEK2, FANCC, FANCM, MLH1, MSH2, MUTYH, NBN, NF1, PALB2, PMS2, PTEN, RAD51C, RAD51D, STK11, TP53, FAM175A, MRE11, RAD51B, RECQL*, and *RINT1* (Nielsen et al., 2016). In this analysis, we identified two patients harboring frameshift indel variants (one in *ATM* and one in *BARD1*) and five patients (including the two sisters) with variants of uncertain clinical significance (VUS) (Table 1). In three patients (MJ2037 and MJ2007/2012) we confirmed the *BRCA1* VUS previously detected by Sanger sequencing. All variants detected in these genes were classified according to the ACMG guidelines (Richards et al., 2015).

**Table 1:** Pathogenic and VUS detected in 27 known HBC genes.

**Patient**	**Gene**	**HGVS nomenclature**	***N* of 6 Damaging**	**dbSNP**	**MAF (ExAC/Abraom)**	**Clinical significance (ClinVar)**	**Clinical significance (ACMG)**
SM001.049	*ATM*	c.7000_7003delTACA; p.(Tyr2334Glnfs^*^4)	**–**	rs786203421	ND/ND	Pathogenic	Pathogenic
MJ1007	*BARD1*	c.2215dupT; p.(Tyr739Leufs^*^2)	**–**	ND	ND/ND	ND	Probably Pathogenic
MJ2003	*RINT1*	c.961T>A; p.(Phe321Ile)	5 of 6	ND	ND/ND	ND	VUS
MJ2001	*RAD51B*	c.728A>G; p.(Lys243Arg)	4 of 6	rs34594234	0.007/0.005	ND	VUS
MJ2037	*BRCA1*	c.5006C>T p.(Ala1669Val)	5 of 6	ND	ND/ND	ND	VUS
MJ2007/2012^#^	*BRCA1*	c.4963T>C; p.(Ser1655Pro)	6 of 6	ND	ND/ND	ND	VUS

# *Sisters; N of 6 Damaging: predictions considered as damaging in 6 pathogenicity predicting software; MAF, minor allele frequency; ND, not described; VUS, variant of unknown clinical significance. RefSeq reference number of transcripts are described at Supplementary Table 2*.

The *ATM* p.(Tyr2334Glnfs^*^4) variant is described as pathogenic in ClinVar database. The *BARD1* p.(Tyr739Leufs^*^2) is not described in any database and was classified as probably pathogenic, since it is a rare truncating variant leading to partial loss of the second BRCT domain and the phosphobinding region. These two variants were confirmed by Sanger sequencing in the proband and, for *ATM*, also in one affected relative (Supplementary Figure 1).

Four rare missense variants identified in our patients were classified as probably damaging by at least four prediction software, and three of them are not described in any population database. Three of them are located in recognized functional domains of the affected proteins: *BRCA1* p.Ala1699Val and p.Ser1655Pro are located at the C-terminal BRCT domain, responsible for BRCA1 interaction with others DNA repair proteins and *RINT1* p.Phe321Ile is located at the functional TIP20 domain.

### Candidate selection for novel breast cancer predisposing genes

Next, for the 15 patients without any probable pathogenic variant (excluding *ATM* and *BARD1* mutated patients) we applied a functional-based variant prioritization. Candidate variants were selected according to the predicted impact in the protein function and affected gene, including all loss-of-function variants (nonsense, frameshift indels, and splice site) as well as missense and in-frame indels occurring in a list of 832 cancer-related genes (DNA repair and cancer related genes—Supplementary Table 1). For the two sisters (MJ2007 and MJ2012), all variants shared between the two were selected as candidates. For the 13 unrelated patients, we selected missense variants predicted to be damaging by at least 4 out of 6 prediction software.

After filtering, we obtained a total of 208 variants, including 125 LOF and 83 missenses (Supplementary Table 2). In order to technically validate our variant selection workflow, a subset of these 208 variants (133 out of 208) was submitted for technical validation by targeted NGS in the same WES samples and, of these, 126 were validated (95%) (Supplementary Table 4). Using this same custom panel, we evaluated 25 control samples of healthy Brazilian women without cancer for filtering common polymorphisms in our population. Eight variants were detected in at least one control sample and where then excluded from our candidates list, resulting in 193 candidate variants (118 validated and 75 not evaluated).

**Figure 2:**
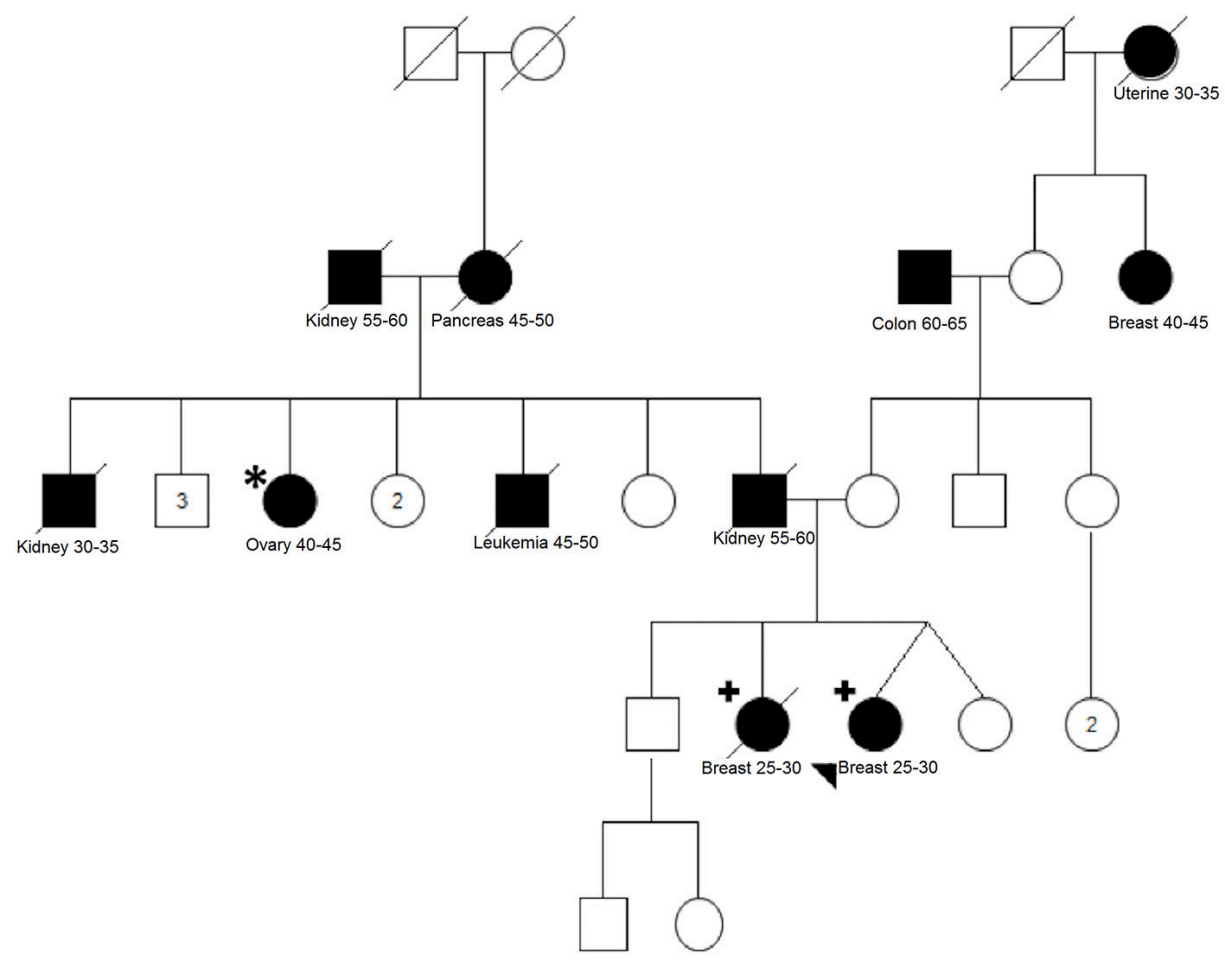
Pedigree of family MJ2007/MJ20012. Two breast cancer affected sisters (indicated with the plus sign) were analyzed using WES. Variants shared between the sisters were screened in one affected paternal aunt (indicated with an asterisk). Filled-in symbols indicate individuals affected by cancer. Cancer type, cancer age of onset or age appear underneath each individual. Numbers inside the symbols indicate the number of unaffected siblings not shown individually in the pedigree.

For the family of the two affected sisters, one additional affected aunt diagnosed with ovarian cancer at age 45 was available for segregation analysis (Figure 2). We analyzed 17 variants that were shared between the two sisters and 8 variants were also present in the aunt, including the VUS variant in *BRCA1* (Table 2).

**Table 2:** Cosegregation analysis of variants detected in the sisters MJ2007 and MJ2012.

**Shared variants of MJ2017 and MJ2012**								**Cosegregation**
**Chr:Pos**	**Ref/Alt**	**Gene names**	**Type**	**HGVS nomenclature**	**N of 6 Damaging**	**dbSNP**	MAF (ExAC^*^/Abraom)	**Present in affected aunt (OV)**
1:3328745	G/A	*PRDM16*	Missense	c.1984G>A; p.(Val662Met)	1 of 6	ND	ND/0.001	No
1:116670844	G/T	*MAB21L3*	Stop gained	c.739G>T; p.(Glu247^*^)	2 of 6	rs149122915	0.0002/ND	No
3:48716158	G/A	*NCKIPSD*	Missense	c.1804C>T; p.(Arg602Cys)	2 of 6	ND	0.000008/0.001	No
5:149514363	A/G	*PDGFRB*	Missense	c.581T>C; p.(Ile194Thr)	5 of 6	rs2229560	0.001/0.002	No
6:117622137	C/T	*ROS1*	Missense	c.6733G>A; p.(Gly2245Ser)	2 of 6	rs142264513	0.0008/0.002	No
7:116380062	A/G	*MET*	Missense	c.1451A>G; p.(His484Arg)	2 of 6	ND	0.00005/ND	No
8:17815082	T/G	*PCM1*	Missense	c.1838T>G; p.(Ile613Ser)	0 of 6	rs181777656	0.003 (OT 0.01)/0.002	No
14:55467701	T/C	*WDHD1*	Missense	c.703A>G; p.(Ile235Val)	1 of 6	rs139440460	0.004/0.004	No
15:40897315	A/G	*KNL1*	Missense	c.43A>G; p.(Ile15Val)	0 of 6	ND	0.00003/ND	No
6:110778048	C/-	*SLC22A16*	Frameshift	c.226delG; p.(Ala76fs^*^66)	–	ND	ND/ND	Yes
6:117715381	A/G	*ROS1*	Missense	c.1108T>C; p.(Ser370Pro)	2 of 6	rs56274823	0.002/ND	Yes
9:6255967	G/C	*IL33*	Splice acceptor	c.613-1G>C; p.(spl?)	–	rs146597587	0.002/0.001	Yes
9:8501026	G/A	*PTPRD*	Missense	c.1856C>T; p.(Thr619Ile)	2 of 6	ND	ND/0.001	Yes
11:120298916	C/T	*ARHGEF12*	Missense	c.545C>T; p.(Ser182Phe)	3 of 6	rs147982337	0.002/0.002	Yes
17:37865694	G/A	*ERBB2*	Missense	c.563G>A; p.(Arg188His)	3 of 6	ND	0.00002/0.002	Yes
17:41222968	A/G	*BRCA1*	Missense	c.4963T>C; p.(Ser1655Pro)	6 of 6	ND	ND/ND	Yes
X:24861673	T/C	*POLA1*	Missense	c.3908T>C; p.(Met1303Thr)	2 of 6	ND	ND/ND	Yes

*Chr, chromosome; Pos, position; Ref, reference allele; Alt, alternate allele; N of 6 Damaging, predictions considered as damaging in 6 pathogenicity predicting software; ND, not described; MAF, minor allele frequency; OV, ovary cancer; OT, others*.

* *Variants in ExAc that had a MAF >1% in any ethnic group are underlined and the highest ExAc population MAF is shown inside parenthesis. RefSeq reference number of transcripts are described at Supplementary Table 2*.

Then, the remaining 186 genes prioritized in our study were compared to candidate genes reported in eight previous WES studies of HBC (Snape et al., 2012; Thompson et al., 2012; Gracia-Aznarez et al., 2013; Hilbers et al., 2013; Kiiski et al., 2014; Wen et al., 2014; Noh et al., 2015; Kim et al., 2017) and 12 common genes were identified, 9 of them presenting LOFs variants in at least one study (Table 3). For two genes the same LOF variants were identified in our and at a second study (*PZP* p.Arg680^*^ and *KRT76* p.Glu276^*^).

**Table 3:** Overlapping genes selected as candidates in other WES studies.

**15 patients (current study)**						**Other studies**				
**Patient**	**Gene**	**Type**	**HGVS nomenclature**	**dbSNP**	MAF (ExAC^*^/Abraom)	**Number/type**	**HGVS nomenclature**	**dbSNP**	MAF (ExAC^*^/Abraom)	**Study**
SM001.040	*NOTCH2*	missense	c.2292T>A; p.(Asn764Lys)	ND	0.00002/ND	2 missense	c.854G>A; p.(Arg285His) c.6178C>T; p.(Arg2060Cys)	rs782452794 rs746551843	0.000008/ND 0.000008/ND	Wen et al., 2014
MJ2013S	*CAPN9*	splice site	c.1657+2T>G; p.(spl?)	rs143145032	0.001/0.002	1 frameshift del	c.1976_1982delAGAATG; p.(Glu659Glyfs^*^20)	ND	0.002/ND	Thompson et al., 2012
MJ2014S and MJ2016S	*DNAH7*	nonsense	c.10359T>G; p.(Tyr3453^*^)	ND	0.000008/ND	1 frameshift ins	c.4787dupA; p.(Tyr1596^*^)	rs573013205	0.002/ND	Thompson et al., 2012
SM001.06	*MST1R*	splice site	c.1231-1G>C; p.(spl?)	ND	0.00008/ND	1 nonsense	c.3322C>T; p.(Arg1108^*^)	rs150876558	0.0001/ND	Thompson et al., 2012
SM001.088	*MSH3*	missense	c.2659G>A; p.(Asp887Asn)	ND	0.00002/ND	1 in frame del	c.162_179del; p.(Ala57_Ala62del)	ND	ND	Thompson et al., 2012
MJ2007/ MJ2012	*ROS1*	missense	c.1108T>C; p.(Ser370Pro)	rs56274823	0.002/ND	1 splice site 1 nonsense	c.5079+2T>C; p.(spl?) c.3303G>A; p.(Trp1101^*^)	ND rs200145587	ND 0.00009/ND	Thompson et al., 2012
MJ2004S	*LAMB4*	splice site	c.34+1G>A; p.(spl?)	rs7788865	0.001 (AF 0.0166)/ 0.003	1 frameshift del	c.5265delA; p.(Lys1755Asnfs^*^11)	rs568834649	0.008/0.004	Thompson et al., 2012
SM001.040	*DDX10*	missense	c.1088G>A; p.(Arg363His)	ND	0.0001/ND	1 nonsense	c.973G>T; p.(Glu325^*^)	ND	ND	Kiiski et al., 2014
MJ2016S	*PZP*	nonsense	c.2038C>T; p.(Arg680^*^)	rs145240281	0.005/ND	1 nonsense	c.2038C>T; p.(Arg680^*^)	rs145240281	0.005 (EU 0.01013)/ ND	Thompson et al., 2012
MJ2015S	*KRT76*	nonsense	c.826G>T; p.(Glu276^*^)	rs149868801	0.007/0.006	1 nonsense	c.826G>T; p.(Glu276^*^)	rs149868801	0.007/0.006	Thompson et al., 2012
MJ2014S	*NIN*	in frame del	c.1736_1738delAAG; p.(Glu579del)	ND	0.0003/0.005	1 frameshift del	c.4261delG; p.(Glu1421Lysfs^*^18)	ND	ND	Thompson et al., 2012
MJ2013S	*SLX4*	missense	c.4766G>A; p.(Arg1589His)	rs746314060	0.00004/0.0008	1 missense	c.2484G>C; p.(Glu828Asp)	rs199656607	0.0001/ND	Kiiski et al., 2014

*ND, not described; MAF, minor allele frequency; AF, Africans; EU, European (Non-Finnish)*.

* *Variants in ExAc that had a MAF >1% in any ethnic group are underlined and the highest ExAc population MAF is shown inside parenthesis. RefSeq reference number of transcripts are described at Supplementary Table 2*.

Thus, from the 193 final candidate variants, we selected 23 candidate genes of BC predisposition: 7 novel candidate genes segregating in the 3 members of the MJ2007/2012 family (S*LC22A16, ROS1, IL33, PTPRD, ARHGEF12, ERBB2, POLA1*), five cancer-related genes harboring LOF variants (*GALNT3, RAF1, PICALM, KL, ERCC1*) and 12 genes overlapping with candidate genes identified in other studies (*CAPN9, KRT76, PZP, DNAH7, MST1R, LAMB4, NIN, MSH3, SLX4, DDX1, NOTCH2*, and *ROS1—ROS1* was also selected in the segregating genes list). The entire coding region of the 23 genes were evaluated in an independent Brazilian cohort.

### Assessing 23 candidate genes in an independent cohort of patients at risk for HBC

To select the most promising candidate genes, we analyzed the 23 candidate genes disclosed in our study in an independent cohort of 42 Brazilian women at risk for HBC. These patients were all negative for pathogenic variants in *BRCA1/2, TP53*, and *ATM* genes. In these data, we assessed the entire coding regions of the selected genes for the presence of rare (MAF < 1%) and possibly pathogenic variants, selecting all LOF variants and missense variants predicted to be pathogenic in at least 3 out of 6 algorithms.

In this cohort, we detected 16 variants in 12 of the 23 candidate genes (Table 4). *NOTCH2* gene was the one with more variants, harboring three missense; *ERBB2* and *DNAH7* harbored two missenses each. Only one LOF variant was detected, affecting *ERCC1* gene, which was the same variant detected in our discovery cohort (c.875G>A; p.Trp292^*^). The remaining genes presented one rare missense variant each.

**Table 4:** Variants detected in 12 candidate genes at the discovery cohort and at an independent cohort.

**15 patients (discovery cohort)**						**42 patients (validation cohort)**					
**Patient**	**Gene**	**Type**	**HGVS nomenclature**	**N of 6 Damaging**	**dbSNP**	MAF (ExAC^*^/Abraom)	**Number/type**	**HGVS nomenclature**	**N of 6 Damaging**	**dbSNP**	**MAF (ExAC/*Abraom*)**
SM001.040	*NOTCH2*	missense	c.2292T>A; p.(Asn764Lys)	6 of 6	ND	0.00002/ND	3 missense	c.2579T>G; p.(Leu860Trp) c.3625T>G; p.(Phe1209Val) c.7223T>A; p.(Leu2408His)	3 of 6 5 of 6 4 of 6	ND rs147223770 rs35586704	ND/ND 0.003/0.002 0.002/0.007
MJ2014S and MJ2016S	*DNAH7*	nonsense	c.10359T>G; p.(Tyr3453^*^)	–	ND	0.000008/ND	2 missense	c.3265C>T; p.(Pro1089Ser) c.11947C>T; p.(Arg3983Trp)	5 of 6 5 of 6	ND rs114621989	ND/ND 0.009/0.006
MJ2016S	*RAF1*	frameshift	c.1241dupA; p.(Asp415fs)	–	ND	ND/ND	1 missense	c.923C>T; p.(Pro308Leu)	3 of 6	rs5746220	0.002/0.008
SM001.06	*MST1R*	splice site	c.1231-1G>C; p.(spl?)	–	ND	0.00008/ND	1 missense	c.4180T>A; p.(Ser1394Thr)	4 of 6	rs141338964	0.0002/ND
MJ2004S	*LAMB4*	splice site	c.34+1G>A; p.(spl?)	–	rs7788865	0.001/0.003	1 missense	c.1843C>A; p.(Pro615Thr)	5 of 6	rs201909531	0.0003/0.0008
MJ2014S	*NIN*	in frame del	c.1736_1738delAAG; p.(Glu579del)	–	ND	0.0003/0.005	1 missense	c.848C>T; p.(Ser283Leu)	3 of 6	rs763293400	0.0001/ND
MJ2013S	*SLX4*	missense	c.4766G>A; p.(Arg1589His)	5 of 6	rs74631406	0.00004/0.0008	1 missense	c.3368C>A; p.(Ser1123Tyr)	3 of 6	rs144647122	0.0003/0.0008
MJ2037S	*ERCC1*	nonsense	c.875G>A; p.(Trp292^*^)	–	rs116640350	0.002 (AF 0.02627)/0.0008	1 nonsense	c.875G>A; p.(Trp292^*^)	–	rs116640350	0.002/0.0008
MJ2007/2012	*SLC22A16*	frameshift	c.226delG; p.(Ala76fs^*^66)	–	ND	ND/ND	1 missense	c.599C>T; p.(Ala200Val)	5 of 6	rs61729086	0.003/0.005
MJ2007/2012	*PTPRD*	missense	c.1856C>T; p.(Thr619Ile)	2 of 6	ND	ND/0.0008	1 missense	c.2585G>T; p.(Arg862Leu)	5 of 6	rs142397137	0.0001/ND
MJ2007/2012	*ARHGEF12*	missense	c.545C>T; p.(Ser182Phe)	3 of 6	rs147982337	0.002/0.002	1 missense	c.3986G>A; p.(Arg1329Gln)	3 of 6	ND	ND/ND
MJ2007/2012	*ERBB2*	missense	c.563G>A; p.(Arg188His)	3 of 6	ND	0.00002/0.002	2 missense	c.236A>C; p.(Glu79Ala) c.1586T>C; p.(Val529Ala)	6 of 6 3 of 6	rs61737968 ND	0.0009/0.005 ND/ND

*N of 6 Damaging, predictions considered as damaging in 6 pathogenicity predicting software; ND, not described; MAF, minor allele frequency; AF, Africans*.

* *Variants in ExAc that had a MAF >1% in any ethnic group are underlined and the highest ExAc population MAF is shown inside parenthesis. RefSeq reference number of transcripts are described at Supplementary Table 4*.

## Discussion

Recently, the use of WES in clinical genetics has been proven to be an effective alternative for establishing the genetic basis of Mendelian diseases, particularly in diseases where multiple genes can be affected (Trujillano et al., 2016). Moreover, in both clinical and research settings, WES has been applied to elucidate the genetic cause of cancer predisposition. In this sense, WES offers the opportunity to concomitantly investigate several known cancer risk genes as well as to identify novel cancer predisposing genes. Thus, in this study we used WES to disclose variants contributing to BC increased risk in patients that were negative for pathogenic variants in three major BC genes—*BRCA1/2* and *TP53* genes—and the most common point mutation in *CHEK2* gene (c.1100delC). For this, we used stringent clinical criteria for selecting patients with strong indicative of harboring a genetic predisposition to BC, such as early onset BC (<36 years); bilateral BC; or the presence of a second primary related tumor.

First, by evaluating known BC predisposing genes, we could establish the causative variants in two probands. One of them harbored an *ATM* truncating pathogenic variant and the other a novel *BARD1* truncating variant, considered as probably pathogenic. The *BARD1* p.(Tyr739Leufs^*^2) variant is predicted to cause partial loss of the second functional BRCT domain and the phosphobinding region. Several studies suggest that both BRCT repeats are necessary for BARD1 normal function (Birrane et al., 2007; Irminger-Finger et al., 2016) and truncating variants in this region have been previously reported in association with HBC (De Brakeleer et al., 2010). Additionally, compatible with the probable pathogenic role of this variant, our proband presented triple negative BC and *BARD1* pathogenic variants were recently described to be related to this molecular subtype (De Brakeleer et al., 2016).

Besides these LOF variants, we identified four rare missense VUS in three HBC genes (*BRCA1, RINT1*, and *RAD51B*). The identification of VUS in genetic testing represent a challenging concern for genetic counselors due to uncertainty in clinical decision making, which can lead to more intensive management than necessary in most of the times or, more rarely, in inappropriate prevention measures (Plon et al., 2011). The recently introduction of NGS gene panels in genetic testing have increased the number of patients diagnosed with VUS, emphasizing the urgent need for better pathogenicity predictions models and collaborative efforts to increase observational data that can aid a posteriori classification to variants, such as cosegregation analysis, personal and family history, co-occurrence with pathogenic variants, and histological and molecular features of tumors (Spurdle et al., 2012).

In the 15 patients without known pathogenic variants, we could identify more than 25,000 novel or rare variants (MAF < 1%), thus several filtering strategies were applied to prioritize those more likely to be related to HBC. Since the majority of hereditary cancer predisposing genes harbor an excess of loss-of function variants, we focused on this type of overtly deleterious variants, regardless of the affected gene. Furthermore, most BC risk genes are involved in DNA repair and genomic integrity pathways (Shiovitz and Korde, 2015; Nielsen et al., 2016), and prioritizing variants in these genes is a rational approach that have been used successfully in previous studies (Mantere et al., 2016). As so, we have also focused on missense variants in a defined set of cancer-related and DNA repair genes. By doing that, we were able to reduce our candidate genes list to a few hundreds.

Importantly, for one family with two sisters affected by BC at young ages (29 years), we could improve the selection by retaining only shared variants and also perform segregation analysis of the candidate variants in an aunt affected by ovarian cancer. From this analysis, eight cosegregating variants emerged, including a *BRCA1* VUS. Besides *BRCA1* gene, only *ERBB2* has been previously implicated in BC predisposition, although with conflicting data about the increased risk conferred by some alleles (Breyer et al., 2009; Wang et al., 2013). Regarding the two LOF variants found to be cosegregating in this family (genes *SLC22A16* and *IL33*), no relation between both genes and BC could be recognized in the literature.

One possible explanation for the results observed in this family and that could also be responsible for the cancer predisposition in other patients of our study is the polygenic model. In this model, which has been suggested and reviewed by different authors (Oldenburg et al., 2007; Shiovitz and Korde, 2015), moderate and low penetrance alleles would act in synergy and play a predominant role. Additionally, the high number of affected relatives with different tumor types in both maternal and paternal sides of this family can be a confounding factor for understanding the phenotypes and cosegregation results. Unfortunately, most affected family members of this family were deceased, limiting additional investigations and the interpretation of our findings.

To gain further insight on the relevance of our identified candidate genes, we evaluated the most promising ones in an independent cohort comprising 42 Brazilian HBC women. Several rare and possibly damaging variants were identified in this cohort, providing additional evidence of the potential role in BC predisposition of some new genes. Of those, we highlight four genes related to cancer development and progression (*NOTCH2, ERBB2, MST1R*, and *RAF1*) and two DNA repair genes (*ERCC1* and *SLX4*). Interestingly, *ERCC1* and *SLX4* are partners that act in the repair of interstrand cross-links and are also required for homology-directed repair of DNA double-strand breaks. Additionally, *ERCC1* is also involved in the nucleotide excision repair pathway (McNeil and Melton, 2012). Both genes have been investigated regarding BC susceptibility, with some common *ERCC1* variants being identified as risk alleles in Chinese population (Yang et al., 2013) and rare truncating and possibly damaging variants in *SLX4* being described in some high risk HBOC patients (Bakker et al., 2013; Shah et al., 2013). Remarkably, in the *ERCC1* gene we identified the same nonsense variant in both discovery and validation cohorts (p.Trp292^*^), while in *SLX4* one of the rare missense identified in our cohorts (p.Ser1123Tyr) was previously described in one HBC patient (Shah et al., 2013).

Some limitations of our study are inherent to WES method since predisposition variants can be located in non-coding or not captured regions of the genome, such as promoter or deep intronic pathogenic variants. Moreover, although the strategic filtering applied here is necessary to reduce the number of proposed candidates, it can result in the omission of the causative variant (for example, by excluding protein-impacting synonymous variants). Additionally, large genomic rearrangements have been implicated in HBC, and even though specific bioinformatics pipelines can be applied in WES data to extract these results, these analyses were not performed in our study. Finally, when it comes to interpreting the potential effect of our candidate variants in splicing, both coding as well as splice site variants can cause splicing alterations that lead to in-frame functional proteins instead of frameshift truncated ones, and functional assays would be necessary to validate bioinformatics predictions.

Considering the evidence presented here, we can neither conclude that these variants identified in the 15 patients negative for known pathogenic variant are the definitive cause of BC predisposition nor determine the magnitude of the risk that these genes could present. Nevertheless, our results provide a set of novel putative BC predisposing genes and reinforce WES as useful tool for assessing the complex landscape of HBC predisposition. Importantly, this represents the first WES data of a HBC cohort from South America and the analysis of an admixed population such as the Brazilian can reveal unique features compared to other Western populations. In this sense, the WES data generated in our study, as well as other previous and future studies, can be reanalyzed in the future and possibly identify genetic overlaps between families, aiding to gene discoveries (Chandler et al., 2016). Finally, the assignment of a novel gene or specific variant as a true BC predisposition factor requires solid phenotypic evidence from cosegregation analysis, *in vitro* and *in vivo* functional assays and genotyping large series of case and controls from distinct populations. The efforts for discovery and validation of novel HBC genes will continue to provide insights into disease mechanisms, eventually leading to the development of more effective therapies and improved management of affected families.

## Author contributions

GT, FdS, EF, and DC: conceived and designed the experiments; GT, FdA, MF, BB, CdP, and EF: performed and analyzed the experiments; RV, JdS, RR, and SdS: performed bioinformatics analysis; AdN, MA, PF, and EP: assessed clinical data, selected, and recruited the patients; SdS, EP, and DC: contributed reagents, materials, and analysis tools; GT, FdA, and DC: wrote and edited the paper. All authors have read and approved the final manuscript.

### Conflict of interest statement

The authors declare that the research was conducted in the absence of any commercial or financial relationships that could be construed as a potential conflict of interest.

## Footnotes

^1^Online Mendelian Inheritance in Man, OMIM®. McKusick-Nathans Institute of Genetic Medicine, Johns Hopkins University (Baltimore, MD), 2018. Available online at: https://omim.org/
