# Supplementary material for: Complex Landscape of Germline Variants in Brazilian Patients With Hereditary and Early Onset Breast Cancer
Source: Front Genet. 2018 May 7;9:161. doi: 10.3389/fgene.2018.00161 (PMC5949367; doi:10.3389/fgene.2018.00161)
Supplement: Supplementary file 1 [file Table_1.DOCX]

**SUPPLEMENTARY DATA**

**Complex landscape of germline variants in hereditary and early onset breast cancer ascertained through whole exome sequencing**

Giovana Tardin Torrezan^*^, Fernanda Gabriella dos Santos Ramos de Almeida^*^, Márcia Cristina Pena Figueiredo, Bruna Durães de Figueiredo Barros, Cláudia A. Andrade de Paula, Renan Valieris, Jorge Estefano Santana de Souza, Rodrigo Fernandes Ramalho, Felipe Cavalcanti Carneiro da Silva, Elisa Napolitano e Ferreira, Amanda França de Nóbrega, Paula Silva Felicio, Maria Isabel Achatz, Sandro José de Souza, Edenir Inêz Palmero, Dirce Maria Carraro

*These authors contributed equally to this study

**Supplementary table 1: 832 genes of interest**

| *ABCC6* | *CD274* | *EPHB6* | *HECW1* | *MCM5* | *PAX2* | *RARA* | *STRA13* |
| --- | --- | --- | --- | --- | --- | --- | --- |
| *ABI1* | *CD74* | *EPS15* | *HELQ* | *MCM6* | *PAX3* | *RB1* | *STX11* |
| *ABL1* | *CD79A* | *ERBB2* | *HERPUD1* | *MCM7* | *PAX5* | *RBBP4* | *STXBP2* |
| *ABL2* | *CD79B* | *ERBB3* | *HES1* | *MDC1* | *PAX6* | *RBBP7* | *SUFU* |
| *ABR* | *CDC20* | *ERBB4* | *HEY1* | *MDM2* | *PAX7* | *RBBP8* | *SUPT3H* |
| *ACSL3* | *CDC20B* | *ERC1* | *HIP1* | *MDM4* | *PAX8* | *RBM15* | *SUZ12* |
| *ACSL6* | *CDC73* | *ERCC1* | *HIST1H2AB* | *MDS2* | *PBRM1* | *RBX1* | *SYK* |
| *ACTR8* | *CDH1* | *ERCC2* | *HIST1H2AC* | *MECOM* | *PBX1* | *RDM1* | *TAF15* |
| *AFF1* | *CDH11* | *ERCC3* | *HIST1H4I* | *MED12* | *PCM1* | *RECQL* | *TAL1* |
| *AFF3* | *CDH6* | *ERCC4* | *HLF* | *MEN1* | *PCNA* | *RECQL4* | *TAL2* |
| *AFF4* | *CDK12* | *ERCC5* | *HLTF* | *MET* | *PCSK7* | *RECQL5* | *TCEA1* |
| *AIP* | *CDK2AP2* | *ERCC6* | *HMGA1* | *MITF* | *PDCD1LG2* | *REL* | *TCEA1P2* |
| *AKAP9* | *CDK4* | *ERCC8* | *HMGA2* | *MKL1* | *PDE4DIP* | *RET* | *TCEA2* |
| *AKT1* | *CDK6* | *ERG* | *HMGB1* | *MLF1* | *PDGFB* | *REV1* | *TCF12* |
| *AKT2* | *CDK7* | *ETV1* | *HMGN2P46* | *MLH1* | *PDGFRA* | *REV3L* | *TCF3* |
| *AKT3* | *CDK8* | *ETV4* | *HNF1A* | *MLH3* | *PDGFRB* | *RFC1* | *TCF4* |
| *ALDH2* | *CDKN1A* | *ETV5* | *HNRNPA2B1* | *MLL* | *PDPK1* | *RFC2* | *TCF7L2* |
| *ALK* | *CDKN1B* | *ETV6* | *HOOK3* | *MLL2* | *PER1* | *RFC3* | *TCL1A* |
| *APC* | *CDKN1C* | *EWSR1* | *HOXA11* | *MLL3* | *PHF6* | *RFC4* | *TCL6* |
| *APEX1* | *CDKN2A* | *EXO1* | *HOXA13* | *MLLT1* | *PHOX2B* | *RFC5* | *TDG* |
| *APEX2* | *CDKN2B* | *EXT1* | *HOXA9* | *MLLT10* | *PIAS1* | *RHBDF2* | *TELO2* |
| *APITD1* | *CDKN2C* | *EXT2* | *HOXB13* | *MLLT11* | *PIAS3* | *RHOH* | *TERC* |
| *APLF* | *CDKN2D* | *EZH2* | *HOXC11* | *MLLT3* | *PIAS4* | *RICTOR* | *TERT* |
| *APTX* | *CDX2* | *FAM123B* | *HOXC13* | *MLLT4* | *PICALM* | *RIF1* | *TET1* |
| *AR* | *CEBPA* | *FAM22A* | *HOXD11* | *MLLT6* | *PIF1* | *RMI1* | *TET2* |
| *ARHGAP26* | *CEP57* | *FAM22B* | *HOXD13* | *MMS19* | *PIK3CA* | *RMI2* | *TFE3* |
| *ARHGEF12* | *CETN2* | *FAM46C* | *HRAS* | *MN1* | *PIK3R1* | *RMND5A* | *TFEB* |
| *ARID1A* | *CHAF1A* | *FAN1* | *HSD17B3* | *MNAT1* | *PIM1* | *RNASEH1* | *TFG* |
| *ARID1B* | *CHAF1B* | *FANCA* | *HSD3B2* | *MNX1* | *PLAG1* | *RNASEH2A* | *TFPT* |
| *ARID2* | *CHCHD7* | *FANCB* | *HSP90AA1* | *MPG* | *PLK1* | *RNASEH2B* | *TFRC* |
| *ARID3A* | *CHD5* | *FANCC* | *HSP90AB1* | *MPL* | *PML* | *RNASEH2C* | *TGFBR1* |
| *ARID3B* | *CHD6* | *FANCD2* | *HSPA14* | *MRE11A* | *PMS1* | *RNASEL* | *TGFBR2* |
| *ARID4A* | *CHEK1* | *FANCE* | *HUS1* | *MSH2* | *PMS2* | *RNF168* | *THRAP3* |
| *ARID4B* | *CHEK2* | *FANCF* | *HUS1B* | *MSH3* | *POLA1* | *RNF8* | *TIMELESS* |
| *ARID5A* | *CHIC2* | *FANCG* | *IDH1* | *MSH6* | *POLA2* | *ROS1* | *TIPIN* |
| *ARID5B* | *CHN1* | *FANCI* | *IDH2* | *MSI2* | *POLB* | *RPA1* | *TLX1* |
| *ARNT* | *CHTF18* | *FANCL* | *IFIH1* | *MSN* | *POLD1* | *RPA2* | *TLX3* |
| *ASPSCR1* | *CIC* | *FANCM* | *IGF1R* | *MTCP1* | *POLD2* | *RPA3* | *TMEM127* |
| *ASXL1* | *CIITA* | *FAS* | *IGHMBP2* | *MTCP1NB* | *POLD3* | *RPA4* | *TMEM30B* |
| *ATAD2* | *CLP1* | *FBXO11* | *IKBKE* | *MTOR* | *POLD4* | *RPL22* | *TMPRSS2* |
| *ATAD5* | *CLSPN* | *FBXO18* | *IKZF1* | *MTUS2* | *POLE* | *RPN1* | *TNFAIP3* |
| *ATF1* | *CLTC* | *FBXW7* | *IL2* | *MUC1* | *POLE2* | *RPTOR* | *TNFRSF14* |
| *ATIC* | *CLTCL1* | *FCGR2B* | *IL21R* | *MUS81* | *POLE3* | *RRM1* | *TNFRSF17* |
| *ATM* | *CNBP* | *FCRL4* | *IL6ST* | *MUTYH* | *POLE4* | *RUNX1* | *TOP1* |
| *ATR* | *CNTRL* | *FEN1* | *IL7R* | *MYB* | *POLG* | *RUNX1T1* | *TOP2A* |
| *ATRIP* | *COL1A1* | *FEV* | *IRF4* | *MYC* | *POLH* | *SARDH* | *TOP3A* |
| *ATRX* | *COX6C* | *FGF23* | *ITK* | *MYCL1* | *POLI* | *SBDS* | *TOP3B* |
| *AURKA* | *CREB1* | *FGFR1* | *JAK1* | *MYCN* | *POLK* | *SDHA* | *TOPBP1* |
| *AXIN2* | *CREB3L1* | *FGFR1OP* | *JAK2* | *MYD88* | *POLL* | *SDHAF2* | *TP53* |
| *BAP1* | *CREB3L2* | *FGFR2* | *JAK3* | *MYH11* | *POLM* | *SDHB* | *TP53BP1* |
| *BARD1* | *CREBBP* | *FGFR3* | *JAZF1* | *MYH9* | *POLN* | *SDHC* | *TPM3* |
| *BCL10* | *CRKL* | *FGFR4* | *JUN* | *MYOC* | *POLQ* | *SDHD* | *TPM4* |
| *BCL11A* | *CRLF2* | *FH* | *KAT6A* | *NACA* | *POU2AF1* | *SEPT5* | *TPR* |
| *BCL11B* | *CRTC1* | *FHIT* | *KAT6B* | *NBN* | *POU5F1* | *SEPT6* | *TRIM24* |
| *BCL2* | *CRTC3* | *FHL1* | *KDM5A* | *NCKIPSD* | *PPARG* | *SEPT9* | *TRIM27* |
| *BCL3* | *CRY1* | *FIP1L1* | *KDM5C* | *NCOA1* | *PPP2R1A* | *SET* | *TRIM33* |
| *BCL6* | *CSF1R* | *FKBP1B* | *KDM6A* | *NCOA2* | *PPP2R5A* | *SETD2* | *TRIP11* |
| *BCL7A* | *CSNK2B* | *FKBP9* | *KDR* | *NCOA4* | *PPP2R5C* | *SETDB1* | *TSC1* |
| *BCL9* | *CSTF2T* | *FLCN* | *KDSR* | *NDRG1* | *PPP2R5D* | *SF3B1* | *TSC2* |
| *BCOR* | *CTNNB1* | *FLI1* | *KEAP1* | *NEIL1* | *PRCC* | *SF3B2* | *TSHR* |
| *BCR* | *CUL3* | *FLT1* | *KIAA1549* | *NEIL2* | *PRDM1* | *SFPQ* | *TTL* |
| *BIRC3* | *CUL4A* | *FLT3* | *KIF1B* | *NEIL3* | *PRDM16* | *SH3GL1* | *TYK2* |
| *ERCC5* | *CUL4B* | *FLT4* | *KIT* | *NF1* | *PRDX3* | *SHFM1* | *U2AF1* |
| *BLID* | *CXCR7* | *FNBP1* | *KL* | *NF2* | *PRDX4* | *SHPRH* | *UBE2NL* |
| *BLM* | *CYLD* | *FOLR1* | *KLF6* | *NFE2L2* | *PRDX5* | *SIRT1* | *UBE2T* |
| *BMPR1A* | *CYP1B1* | *FOXC1* | *KLK2* | *NFIB* | *PRF1* | *SIRT5* | *UBR1* |
| *BRAF* | *DAXX* | *FOXL2* | *KRAS* | *NFKB2* | *PRIM1* | *SLC25A39* | *UBR2* |
| *BRCA1* | *DCLRE1C* | *FOXO1* | *KRT17* | *NHEJ1* | *PRIM2* | *SLC45A3* | *UNC13D* |
| *BRCA2* | *DDB1* | *FOXO3* | *KTN1* | *NIN* | *PRKAR1A* | *SLX1A* | *UNG* |
| *BRD3* | *DDB2* | *FOXO4* | *LASP1* | *NKX2-1* | *PRKD1* | *SLX1B* | *USP1* |
| *BRD4* | *DDIT3* | *FOXP1* | *LCK* | *NONO* | *PRKD2* | *SLX4* | *USP6* |
| *BRIP1* | *DDX10* | *FSTL3* | *LCP1* | *NOTCH1* | *PRKD3* | *SMAD2* | *UTY* |
| *BRSK1* | *DDX11* | *FUBP1* | *LHFP* | *NOTCH2* | *PRKDC* | *SMAD3* | *VHL* |
| *BRSK2* | *DDX5* | *FUS* | *LIFR* | *NOTCH3* | *PRRC2A* | *SMAD4* | *VPS53* |
| *BTG1* | *DDX58* | *FZR1* | *LIG1* | *NOTCH4* | *PRRC2C* | *SMARCA4* | *VTI1A* |
| *BUB1* | *DDX6* | *GALNT12* | *LIG3* | *NPM1* | *PRRX1* | *SMARCA5* | *WAPAL* |
| *BUB1B* | *DEK* | *GALNT3* | *LIG4* | *NPRL3* | *PSIP1* | *SMARCB1* | *WAS* |
| *BUB3* | *DHX58* | *GAS7* | *LMO1* | *NR4A3* | *PSMD4* | *SMARCE1* | *WDHD1* |
| *C15orf55* | *DICER1* | *GATA1* | *LMO2* | *NRAS* | *PTCH1* | *SMC6* | *WDR36* |
| *C17orf70* | *DIS3L2* | *GATA2* | *LPP* | *NSD1* | *PTEN* | *SMG5* | *WDR48* |
| *C19orf40* | *DNA2* | *GATA3* | *LRP5* | *NTHL1* | *PTK2* | *SMG6* | *WDR73* |
| *CAMKK1* | *DNAJA4* | *GEN1* | *LTBP2* | *NTRK1* | *PTK2B* | *SMG7* | *WHSC1* |
| *CAMKK2* | *DNMT3A* | *GLMN* | *LTBP3* | *NTRK2* | *PTPN11* | *SMO* | *WHSC1L1* |
| *CANT1* | *DNTT* | *GMPS* | *LYL1* | *NTRK3* | *PTPRD* | *SMUG1* | *WIF1* |
| *CARD11* | *DSCC1* | *GNA11* | *MAD2L1BP* | *NUMA1* | *RABEP1* | *SNX29* | *WRN* |
| *CARS* | *DUX4* | *GNAQ* | *MAD2L2* | *NUP107* | *RAD1* | *SOCS1* | *WT1* |
| *CASC5* | *EBF1* | *GNAS* | *MAF* | *NUP133* | *RAD17* | *SOX2* | *XAB2* |
| *CASR* | *EGFR* | *GOLGA5* | *MAFB* | *NUP153* | *RAD18* | *SPECC1* | *XPA* |
| *CBFA2T3* | *EIF4A2* | *GOPC* | *MALAT1* | *NUP214* | *RAD23A* | *SPEN* | *XPC* |
| *CBFB* | *ELAC2* | *GPC3* | *MALT1* | *NUP98* | *RAD23B* | *SPRED1* | *XPO1* |
| *CBL* | *ELF4* | *GPHN* | *MAML2* | *OGG1* | *RAD50* | *SRC* | *XRCC1* |
| *CBLB* | *ELK4* | *GREM1* | *MAP2K1* | *OLIG2* | *RAD51* | *SRD5A2* | *XRCC2* |
| *CBLC* | *ELL* | *GSTM1* | *MAP2K2* | *OMD* | *RAD51B* | *SRGAP3* | *XRCC3* |
| *CCDC6* | *ELN* | *GTF2H1* | *MAP2K4* | *OPTN* | *RAD51C* | *SRSF2* | *XRCC4* |
| *CCNB1* | *EME1* | *GTF2H2* | *MAP3K1* | *P2RY8* | *RAD51D* | *SRSF3* | *XRCC5* |
| *CCNB1IP1* | *EME2* | *GTF2H2C* | *MAP3K8* | *PAFAH1B2* | *RAD52* | *SS18* | *XRCC6* |
| *CCNB2* | *EML4* | *GTF2H2C_2* | *MAX* | *PAK7* | *RAD54B* | *SS18L1* | *YWHAE* |
| *CCND1* | *ENG* | *GTF2H3* | *MBD4* | *PALB2* | *RAD54L* | *SSBP1* | *ZBTB16* |
| *CCND2* | *EP300* | *GTF2H4* | *MC1R* | *PALLD* | *RAD9A* | *SSX1* | *ZMYM2* |
| *CCND3* | *EPCAM* | *GTF2H5* | *MCL1* | *PARP1* | *RAD9B* | *SSX2* | *ZNF331* |
| *CCNE1* | *EPHA10* | *GUCY1A2* | *MCM2* | *PARP2* | *RAF1* | *SSX4* | *ZNF384* |
| *CCNH* | *EPHA3* | *H2AFJ* | *MCM3* | *PARP3* | *RALGDS* | *STAT3* | *ZNF521* |
| *CCNT1* | *EPHA5* | *HDAC3* | *MCM3AP* | *PARP4* | *RANBP17* | *STIL* | *ZNF668* |
| *CCNT2* | *EPHA6* | *HDAC8* | *MCM4* | *PATZ1* | *RAP1GDS1* | *STK11* | *ZRSR2* |

**Supplementary table 2: Final candidate selection and results of validation stages**

| **Chr:Pos** | **Ref/Alt** | **Gene** | **Sequence Ontology** | **Transcript** | **HGVS nomenclature** | **Target validation** | **Presence in CTLs** | **dbSNP** | **ExAC**  **MAF** | **ABraOm MAF** | **Zigosity** | **Sample** |
| --- | --- | --- | --- | --- | --- | --- | --- | --- | --- | --- | --- | --- |
| 1:100626087 | GCTTGGT/- | LRRC39 | stop_gained | NM_001256385.1 | c.148_154delACCAAGC; p.Thr50Ter | VAL | N | ND | 0.0003624 | 0.001 | ht | MJ2016S/  MJ2037S |
| 1:110200294 | G/A | GSTM4 | splice_donor | NM_000850.4 | c.259+1G>A; p.spl? | NE | NE | rs41283498 | 0.001656 | 0.002 | ht | MJ1011S |
| 1:114377567 | C/T | PTPN22 | stop_gained | NM_015967.5 | c.1859G>A; p.Trp620Ter | VAL | N | ND | 0.0003047 | ND | ht | MJ2001S |
| 1:116670844 | G/T | MAB21L3 | stop_gained | NM_152367.2 | c.739G>T; p.Glu247Ter | VAL | N | rs149122915 | 0.0001977 | ND | ht | MJ2007S/  MJ2012S |
| 1:120496239 | A/T | NOTCH2 | missense | NM_024408.3 | c.2292T>A; p.Asn764Lys | NE | NE | ND | 0.00001647 | ND | ht | SM001_040 |
| 1:144922215 | A/G | PDE4DIP | missense | NM_001198834.3 | c.953T>C; p.Leu318Pro | VAL | N | ND | 0.00001647 | 0.001 | ht | MJ2037S |
| 1:145561822 | C/T | ANKRD35 | stop_gained | NM_144698.4 | c.1510C>T; p.Arg504Ter | VAL | N | rs139177747 | **0.001969 / EU 0.02022** | 0.002 | ht | MJ2037S |
| 1:152191806 | G/A | HRNR | stop_gained | NM_001009931.2 | c.2299C>T; p.Arg767Ter | VAL | Y | rs148459733 | **0.007 /**  **LA 0.07922** | 0.007 | ht | MJ2014S |
| 1:156786487 | A/T | SH2D2A | missense | N001161441.1 | c.14T>A; p.Leu5Gln | VAL | N | rs142614264 | 0.001475 | 0.007 | ht | SM001_088 |
| 1:15987960 | ACAG/- | RSC1A1 | frameshift | NM_006511.1 | c.1597_1600delACAG; p.Asp534fs | VAL | N | ND | 0.00007413 | ND | ht | SM001_068 |
| 1:16259648 | AGC/- | SPEN | inframe_deletion | NM_015001.2 | c.6913_6915delAGC; p.Ser2306del | NE | NE | ND | **0.005543 /** **LA 0.03875** | 0.002 | ht | MJ2014S/  SM001_049 |
| 1:19018305 | G/A | PAX7 | missense | NM_002584.2 | c.644G>A; p.Arg215His | VAL | N | rs200575057 | 0.0004616 | ND | ht | SM001_068 |
| 1:196928157 | C/T | CFHR2 | stop_gained | NM_005666.2 | c.760C>T; p.Arg254Ter | VAL | N | rs41313888 | 0.0007414 | 0.003 | ht | MJ2015S |
| 1:196967280 | C/A | CFHR5 | stop_gained | NM_030787.3 | c.993C>A; p.Cys331Ter | VAL | N | ND | 0.00005872 | ND | ht | SM001_068 |
| 1:203186975 | T/A | CHIT1 | stop_gained | NM_003465.2 | c.1048A>T; p.Lys350Ter | VAL | N | ND | ND | ND | ht | MJ2014S |
| 1:230925937 | T/G | CAPN9 | splice_donor | NM_006615.2 | c.1657+2T>G; p.spl? | NE | NE | rs143145032 | 0.0009554 | 0.002 | ht | MJ2013S |
| 1:247320105 | G/T | ZNF124 | stop_gained | NM_003431.2 | c.633C>A; p.Tyr211Ter | VAL | N | rs33998996 | **0.00453 / FN 0.01331** | 0.005 | ht | SM001_068 |
| 1:3328745 | G/A | PRDM16 | missense | NM_022114.3 | c.1984G>A; p.Val662Met | NE | NE | ND | ND | 0.001 | ht | MJ2007S/  MJ2012S |
| 1:35227239 | G/A | GJB4 | stop_gained | NM_153212.2 | c.384G>A; p.Trp128Ter | VAL | N | rs149110828 | 0.002109 | 0.001 | ht | MJ2015S |
| 1:3672032 | G/T | CCDC27 | stop_gained | NM_152492.2 | c.454G>T; p.Glu152Ter | VAL | N | rs139820400 | 0.00008236 | ND | ht | MJ2016S |
| 1:46726525 | C/T | RAD54L | missense | NM_003579.3 | c.604C>T; p.Arg202Cys | NE | NE | rs28363218 | 0.002924 | 0.007 | ht | MJ2013S |
| 1:63064446 | A/- | ANGPTL3 | frameshift | NM_014495.3 | c.575delA; p.Gln192fs | VAL | N | ND | 0.0001648 | ND | ht | SM001_040 |
| 1:6639188 | G/- | TAS1R1 | frameshift | NM_138697.3 | c.2070delG; p.Gln690fs | VAL | N | ND | 0.0003871 | ND | ht | MJ1011S |
| 1:75185057 | C/T | CRYZ | splice_acceptor | NM_001889.3 | c.265-1G>A; p.spl? | NE | NE | rs139734636 | 0.0007909 | 0.001 | ht | MJ2004S |
| 1:94508360 | G/T | ABCA4 | stop_gained | NM_000350.2 | c.3285C>A; p.Tyr1095Ter | VAL | N | ND | ND | ND | ht | SM001_040 |
| 10:102059412 | A/C | PKD2L1 | stop_gained | NM_016112.2 | c.413T>G; p.Leu138Ter | VAL | N | rs200474106 | 0.0001977 | 0.002 | ht | MJ1011S |
| 10:111640599 | C/T | XPNPEP1 | splice_donor | NM_020383.3 | c.1131+1G>A; p.spl? | NE | NE | rs376329109 | 0.000008 | ND | ht | SM001_040 |
| 10:127458937 | C/T | MMP21 | stop_gained | NM_147191.1 | c.1203G>A; p.Trp401Ter | VAL | N | rs137955225 | 0.00003295 | ND | ht | MJ2037S |
| 10:14974896 | G/A | DCLRE1C | missense | NM_001033855.1 | c.737C>T; p.Thr246Ile | NE | NE | rs374596045 | 0.00008236 | ND | ht | SM001_088 |
| 10:38260638 | G/A | ZNF25 | stop_gained | NM_145011.2 | c.13C>T; p.Gln5Ter | VAL | N | ND | ND | ND | ht | MJ2037S |
| 10:84744882 | C/T | NRG3 | stop_gained | NM_001010848.3 | c.1612C>T; p.Arg538Ter | VAL | N | ND | 0.000008 | ND | ht | MJ2004S |
| 10:88681437 | C/T | BMPR1A | missense | NM_004329.2 | c.1327C>T; p.Arg443Cys | VAL | N | rs35619497 | 0.0006095 | 0.002 | ht | MJ2037S |
| 10:90574981 | G/- | LIPM | frameshift | NM_001128215.1 | c.711delG; p.Gly238fs | VAL | N | rs138170497 | **0.003215 / FN 0.01205** | 0.002 | ht | SM001_021 |
| 10:99338075 | G/T | ANKRD2 | stop_gained | NM_020349.2 | c.349G>T; p.Glu117Ter | VAL | N | rs373751289 | 0.00005767 | ND | ht | MJ2037S |
| 11:102586146 | G/A | MMP8 | stop_gained | NM_002424.2 | c.925C>T; p.Gln309Ter | VAL | N | ND | 0.000008 | 0.003 | ht | MJ2004S |
| 11:108175463 | A/T | ATM | missense | NM_000051.3 | c.5558A>T; p.Asp1853Val | VAL | N | rs1801673 | 0.005164 | 0.007 | ht | MJ2003S |
| 11:108352074 | CT/- | KDELC2 | frameshift | NM_153705.4 | c.930_931delAG; p.Arg310fs | VAL | N | ND | 0.0008859 | 0.003 | ht | MJ2016S |
| 11:108562715 | G/A | DDX10 | missense | NM_004398.2 | c.1088G>A; p.Arg363His | NE | NE | ND | 0.0001483 | ND | ht | SM001_040 |
| 11:112050123 | C/T | BCO2 | stop_gained | NM_031938.5 | c.211C>T; p.Arg71Ter | VAL | N | ND | 0.0001235 | 0.001 | ht | MJ2014S |
| 11:116660952 | TGTT/- | APOA5 | frameshift | NM_052968.4 | c.990_993delAACA; p.Asp332fs | VAL | N | ND | 0.000008 | ND | ht | SM001_040 |
| 11:120298916 | C/T | ARHGEF12 | missense | NM_015313.2 | c.545C>T; p.Ser182Phe | NE | NE | rs147982337 | 0.001672 | 0.002 | ht | MJ2007S/  MJ2012S |
| 11:18741652 | T/A | IGSF22 | splice_acceptor | NM_173588.3 | c.479-2A>T; p.spl? | NE | NE | rs201892326 | 0.002218 | 0.001 | ht | MJ2001S |
| 11:3697789 | C/T | NUP98 | missense | NM_016320.4 | c.5135G>A; p.Arg1712Gln | VAL | N | rs146972892 | 0.000173 | 0.001 | ht | SM001_040 |
| 11:44148399 | C/T | EXT2 | missense | NM_000401.3 | c.1072C>T; p.Arg358Trp | VAL | N | rs145611597 | 0.00002471 | ND | ht | SM001_040 |
| 11:4593488 | AG/- | C11orf40 | frameshift | NM_144663.1 | c.344_345delCT; p.Ser115fs | VAL | Y | ND | 0.0003 | ND | ht | MJ2004S |
| 11:61205313 | C/A | SDHAF2 | missense | NM_017841.2 | c.253C>A; p.Leu85Ile | NE | NE | ND | ND | ND | ht | SM001_040 |
| 11:618112 | C/T | CDHR5 | splice_acceptor | NM_021924.4 | c.1961-1G>A; p.spl? | NE | NE | ND | ND | ND | ht | SM001_040 |
| **11:85737334** | **C/A** | **PICALM** | **stop_gained#** | **NM_007166.3** | **c.349G>T; p.Gly117Ter** | **VAL** | **N** | **ND** | **ND** | **ND** | **ht** | **MJ2014S** |
| 11:94211916 | C/T | MRE11A | missense | NM_005591.3 | c.529G>A; p.Ala177Thr | VAL | N | rs142996063 | 0.0002059 | 0.001 | ht | MJ2014S |
| 12:10977956 | TG/- | TAS2R10 | frameshift | NM_023921.1 | c.912_913delCA; p.Arg305fs | VAL | N | ND | 0.0006343 | 0.001 | ht | SM001_068 |
| 12:124179417 | T/- | TCTN2 | frameshift | NM_024809.4 | c.1128delT; p.Arg377fs | NE | NE | ND | ND | ND | ht | MJ2015S |
| 12:31256907 | C/A | DDX11 | stop_gained | NM_152438.1 | c.2853C>A; p.Cys951Ter | NVal | N | ND | **0.003 /**  **AF 0.03243** | ND | ht | MJ2037S |
| 12:44142401 | G/A | PUS7L | stop_gained | NM_031292.4 | c.922C>T; p.Arg308Ter | VAL | N | rs141745509 | 0.000313 | 0.002 | ht | MJ2013S |
| 12:52711549 | G/T | KRT83 | stop_gained | NM_002282.3 | c.666C>A; p.Cys222Ter | NE | NE | rs2857667 | 0.005164 | ND | ht | MJ2004S |
| 12:53167416 | C/A | KRT76 | stop_gained | NM_015848.4 | c.826G>T; p.Glu276Ter | VAL | N | rs149868801 | 0.00663 | 0.007 | ht | MJ2015S |
| 12:57441459 | G/A | MYO1A | stop_gained | NM_005379.3 | c.277C>T; p.Arg93Ter | VAL | N | rs121909305 | 0.003204 | ND | ht | MJ2016S |
| 12:9321534 | G/A | PZP | stop_gained | NM_002864.2 | c.2038C>T; p.Arg680Ter | VAL | N | rs145240281 | **0.005395 / EU 0.01013** | ND | ht | MJ2016S |
| 13:25265101 | G/C | ATP12A | splice_acceptor | NM_001185085.1 | c.800-1G>C; p.spl? | NE | NE | rs114726296 | 0.0005683 | 0.002 | ht | MJ2004S |
| 13:33638317 | C/A | KL | stop_gained | NM_004795.3 | c.3033C>A; p.Tyr1011Ter | VAL | N | ND | ND | 0.001 | ht | SM001_021 |
| 13:46619201 | T/A | ZC3H13 | splice_acceptor | NM_015070.3 | c.118-2A>T; p.spl? | NE | NE | ND | ND | ND | ht | MJ2016S |
| 13:46708276 | T/C | LCP1 | missense | NM_002298.4 | c.1612A>G; p.Ile538Val | NE | NE | rs148900496 | 0.0001071 | 0.001 | ht | MJ2037S |
| 13:49033954 | C/G | RB1 | missense | NM_000321.2 | c.2091C>G; p.Asp697Glu | VAL | N | rs3092903 | 0.000008 | ND | ht | MJ2037S |
| 14:50117066 | A/G | POLE2 | missense | NM_002692.3 | c.1414T>C; p.Tyr472His | NE | NE | rs34000915 | **0.004406 / EU 0.02904** | 0.003 | ht | SM001_088 |
| 14:51230580 | CTT/- | NIN | inframe_deletion | NM_020921.3 | c.1736_1738delAAG; p.Glu579del | NE | NE | ND | 0.0002883 | 0.005 | ht | MJ2014S |
| 14:55467701 | T/C | WDHD1 | missense | NM_007086.3 | c.703A>G; p.Ile235Val | NE | NE | rs139440460 | 0.003591 | 0.004 | ht | MJ2007S/  MJ2012S |
| 14:56108429 | AA/TC | KTN1 | missense | NM_001079521.1 | c.2123_2124delinsTC; p.Lys708Ile | NE | NE | rs140464911 | 0.0003872 | 0.001 | ht | MJ2037S/  SM001_040 |
| 14:68053899 | C/T | PLEKHH1 | stop_gained | NM_020715.2 | c.4042C>T; p.Arg1348Ter | VAL | N | rs111462449 | **0.007569 / EU 0.01149** | 0.006 | ht | MJ2014S/  SM001_068 |
| 14:68353893 | A/G | RAD51B | missense | NM_133509.3 | c.728A>G; p.Lys243Arg | NE | NE | rs34594234 | **0.00733 / EU 0.01068** | 0.005 | ht | MJ2001S |
| 14:74976838 | C/A | LTBP2 | missense | NM_000428.2 | c.3107G>T; p.Cys1036Phe | NE | NE | rs374444394 | 0.00004118 | ND | ht | MJ2037S |
| 14:75514489 | C/G | MLH3 | missense | NM_001040108.1 | c.1870G>C; p.Glu624Gln | NE | NE | rs28756986 | **0.007281 / EU 0.01068** | 0.009 | ht | SM001_088 |
| 15:40897315 | A/G | CASC5 | missense | NM_170589.4 | c.43A>G; p.Ile15Val | VAL | N | ND | 0.00003312 | ND | ht | MJ2007S/  MJ2012S |
| 15:41796352 | C/A | LTK | stop_gained | NM_002344.5 | c.2437G>T; p.Glu813Ter | VAL | Y | rs148281714 | 0.004 | 0.004 | ht | SM001_040 |
| 15:42169382 | G/A | SPTBN5 | stop_gained | NM_016642.3 | c.3643C>T; p.Arg1215Ter | VAL | N | ND | 0.00001651 | ND | ht | SM001_068 |
| 15:63970421 | G/C | HERC1 | stop_gained | NM_003922.3 | c.6693C>G; p.Tyr2231Ter | VAL | N | ND | ND | ND | ht | SM001_068 |
| 15:91184414 | C/T | CRTC3 | missense | NM_022769.4 | c.1634C>T; p.Pro545Leu | NE | NE | ND | 0.00005765 | ND | ht | MJ2013S |
| 16:21181827 | -/C | TMEM159 | frameshift | NM_020422.4 | c.165_166insC; p.Leu56fs | VAL | N | ND | 0.00004942 | 0.002 | ht | SM001_040 |
| 16:2130352 | C/T | TSC2 | missense | NM_000548.3 | c.3584C>T; p.Ala1195Val | VAL | N | ND | 0.000008 | ND | ht | MJ2014S |
| 16:320992 | C/A | RGS11 | stop_gained | NM_183337.1 | c.970G>T; p.Glu324Ter | VAL | N | rs61759901 | **0.006227 / EU 0.01091** | 0.006 | ht | SM001_040 |
| 16:3633485 | C/T | SLX4 | missense | NM_032444.2 | c.4766G>A; p.Arg1589His | VAL | N | ND | 0.00004118 | 0.001 | ht | MJ2013S |
| 16:57016150 | G/A | CETP | splice_donor | NM_000078.2 | c.1321+1G>A; p.spl? | NE | NE | ND | 0.00003295 | ND | ht | SM001_040 |
| 16:89986130 | T/C | MC1R | missense | NM_002386.3 | c.464T>C; p.Ile155Thr | NE | NE | rs1110400 | 0.005234 | 0.006 | ht | MJ2003S |
| 17:28380800 | AG/- | EFCAB5 | frameshift | NM_198529.3 | c.1828_1829delAG; p.Glu611fs | VAL | N | ND | 0.0004792 | ND | ht | MJ2014S |
| 17:36482604 | AG/- | GPR179 | frameshift | NM_001004334.2 | c.6847_6848delCT; p.Leu2283fs | VAL | N | ND | 0.00001655 | ND | ht | SM001_040 |
| 17:37863339 | A/G | ERBB2 | missense | NM_004448.2 | c.170A>G; p.Gln57Arg | VAL | N | rs140441229 | 0.0001236 | 0.001 | ht | SM001_040 |
| 17:37865694 | G/A | ERBB2 | missense | NM_004448.2 | c.563G>A; p.Arg188His | VAL | N | ND | 0.00002471 | 0.002 | ht | MJ2007S/  MJ2012S |
| 17:41219693 | G/A | BRCA1 | missense | NM_007294.3 | c.5006C>T; p.Ala1669Val | VAL | N | ND | ND | ND | ht | MJ2037S |
| 17:41222968 | A/G | BRCA1 | missense | NM_007294.3 | c.4963T>C; p.Ser1655Pro | VAL | N | ND | ND | ND | ht | MJ2007S/  MJ2012S |
| 17:42399875 | T/C | SLC25A39 | missense | NM_001143780.1 | c.236A>G; p.Asn79Ser | NE | NE | rs199503270 | 0.0002224 | ND | ht | MJ2003S |
| 17:56772522 | G/A | RAD51C | missense | NM_058216.2 | c.376G>A; p.Ala126Thr | NE | NE | rs61758784 | 0.003476 | 0.007 | ht | SM001_021 |
| 17:66538223 | C/T | FAM20A | missense | NM_017565.3 | c.1012G>A; p.Gly338Ser | VAL | Y | rs35871101 | 0.0004 | ND | ht | MJ2001S |
| 17:67190524 | A/G | ABCA10 | splice_donor | NM_080282.3 | c.1345+2T>C; p.spl? | NE | NE | ND | 0.00003295 | ND | ht | MJ1011S |
| 17:76161969 | G/T | C17orf99 | splice_acceptor | NM_001163075.1 | c.641-1G>T; p.spl? | NE | NE | rs181766377 | 0.0002321 | 0.001 | ht | MJ2014S |
| 17:78866559 | G/A | RPTOR | missense | NM_020761.2 | c.2132G>A; p.Ser711Asn | NE | NE | ND | ND | 0.004 | ht | MJ1011S |
| 18:56376784 | A/G | MALT1 | missense | NM_006785.3 | c.824A>G; p.Tyr275Cys | VAL | N | rs145169756 | 0.0001565 | ND | ht | MJ2004S |
| 19:16272284 | G/A | CIB3 | stop_gained | NM_054113.2 | c.556C>T; p.Arg186Ter | VAL | N | ND | 0.00001647 | ND | ht | SM001_040 |
| 19:17339687 | C/T | OCEL1 | stop_gained | NM_024578.1 | c.748C>T; p.Gln250Ter | VAL | N | rs147139447 | 0.0002471 | ND | ht | MJ2013S |
| 19:36003591 | G/T | DMKN | stop_gained | NM_033317.4 | c.528C>A; p.Tyr176Ter | VAL | N | rs78947561 | 0.0002553 | 0.001 | ht | MJ2004S |
| 19:36250842 | G/A | C19orf55 | stop_gained | NM_001039887.2 | c.261G>A; p.Trp87Ter | VAL | N | rs116498218 | **0.001829 / AF 0.03448** | 0.005 | ht | SM001_021 |
| 19:45916903 | C/T | ERCC1 | stop_gained | NM_202001.2 | c.875G>A; p.Trp292Ter | VAL | N | rs116640350 | **0.002479 / AF 0.02627** | 0.001 | ht | MJ2037S |
| 19:58118499 | C/T | ZNF530 | stop_gained | NM_020880.3 | c.1606C>T; p.Gln536Ter | NVal | N | rs150850917 | 0.00002 | 0.002 | ht | MJ2003S |
| 19:6222553 | G/A | MLLT1 | missense | NM_005934.3 | c.689C>T; p.Ser230Leu | NE | NE | rs146940691 | 0.0007916 | 0.004 | ht | SM001_040 |
| 19:6426574 | T/C | SLC25A41 | splice_acceptor | NM_173637.3 | c.941-2A>G; p.spl? | NE | NE | rs115373700 | **0.001637 / AF 0.01893** | 0.005 | ht | SM001_040 |
| 19:8922166 | A/- | ZNF558 | frameshift | NM_144693.1 | c.1000delT; p.Ser334fs | NE | NE | ND | ND | ND | ht | SM001_040 |
| 2:135745373 | G/A | MAP3K19 | stop_gained | NM_025052.3 | c.1069C>T; p.Arg357Ter | VAL | Y | ND | 0.000008 | ND | ht | SM001_088 |
| 2:163123994 | C/T | IFIH1 | missense | NM_022168.3 | c.2893G>A; p.Gly965Ser | NE | NE | rs202009944 | 0.00008237 | ND | ht | SM001_088 |
| 2:166626727 | G/A | GALNT3 | stop_gained | NM_004482.3 | c.484C>T; p.Arg162Ter | VAL | N | rs137853086 | 0.0001153 | ND | ht | MJ2014S |
| 2:169688033 | C/T | NOSTRIN | stop_gained | NM_001171631.1 | c.394C>T; p.Gln132Ter | VAL | N | rs372867936 | 0.000008 | ND | ht | MJ2014S |
| 2:176958235 | A/G | HOXD13 | missense | NM_000523.3 | c.617A>G; p.Tyr206Cys | NE | NE | rs147720746 | 0.001054 | 0.001 | ht | SM001_021 |
| 2:190617412 | -/A | OSGEPL1 | frameshift | NM_022353.2 | c.1133dupT; p.Leu378fs | NE | NE | ND | 0.002 | 0.003 | ht | MJ1007S/  MJ2003S |
| 2:196661456 | A/C | DNAH7 | stop_gained | NM_018897.2 | c.10359T>G; p.Tyr3453Ter | VAL | N | ND | 0.000008 | ND | ht | MJ2014S/  MJ2016S |
| 2:209025758 | G/A | CRYGA | stop_gained | NM_014617.3 | c.295C>T; p.Arg99Ter | VAL | N | rs116344874 | **0.002693 / LA 0.02706** | 0.003 | ht | SM001_040 |
| 2:210880731 | G/A | RPE | stop_gained | NM_199229.2 | c.237G>A; p.Trp79Ter | VAL | N | rs375232666 | 0.00004942 | ND | ht | MJ2015S |
| 2:223559079 | A/G | MOGAT1 | splice_acceptor | NM_058165.2 | c.479-2A>G; p.spl? | NE | NE | rs202012168 | 0.0006457 | 0.001 | ht | MJ2037S |
| 2:27745372 | C/T | GCKR | stop_gained | NM_001486.3 | c.1618C>T; p.Arg540Ter | VAL | N | rs146053779 | 0.0008813 | ND | ht | MJ2014S |
| 2:27849929 | TC/- | CCDC121 | frameshift | NM_001142683.2 | c.1223_1224delGA; p.Arg408fs | NVal | N | ND | ND | ND | ht | MJ2015S |
| 2:27905207 | T/A | SLC4A1AP | stop_gained | NM_018158.2 | c.1856T>A; p.Leu619Ter | NVal | N | ND | ND | ND | ht | MJ2004S |
| 2:31756451 | C/A | SRD5A2 | missense | NM_000348.3 | c.536G>T; p.? | NE | NE | ND | 0.00004153 | ND | ht | SM001_088 |
| 2:39216456 | C/T | SOS1 | splice_acceptor | NM_005633.3 | c.3347-1G>A; p.spl? | NE | NE | rs141565234 | 0.0001236 | 0.002 | ht | MJ2014S |
| 2:86992263 | A/C | RMND5A | missense | NM_022780.3 | c.635A>C; p.Glu212Ala | NE | NE | ND | ND | 0.001 | ht | MJ2003S |
| 2:99634688 | G/A | TSGA10 | stop_gained | NM_025244.2 | c.2047C>T; p.Arg683Ter | VAL | N | rs368644089 | 0.00004118 | ND | ht | MJ2013S |
| 20:257724 | A/- | C20orf96 | frameshift | NM_153269.2 | c.786delT; p.Gln263fs | VAL | N | ND | 0.0007001 | 0.006 | ht | MJ2037S |
| 20:36989357 | G/T | LBP | splice_acceptor | NM_004139.3 | c.589-1G>T; p.spl? | NE | NE | rs140824493 | 0.0001318 | ND | ht | MJ1011S |
| 20:40980873 | T/A | PTPRT | missense | NM_133170.3 | c.1613A>T; p.Gln538Leu | VAL | N | rs372526838 | 0.00004964 | ND | ht | MJ1011S |
| 20:58557976 | A/G | CDH26 | splice_acceptor | NM_177980.2 | c.394-2A>G; p.spl? | NE | NE | rs201613457 | 0.0001647 | ND | ht | SM001_021 |
| 21:31655155 | A/- | KRTAP24-1 | frameshift | NM_001085455.1 | c.96delT; p.Thr33fs | VAL | N | ND | 0.002399 | 0.002 | ht | SM001_088 |
| 22:29693915 | G/A | EWSR1 | missense | NM_013986.3 | c.1408G>A; p.Gly470Ser | NE | NE | rs41311143 | **0.008228 / EU 0.01321** | 0.005 | ht | MJ2003S |
| 22:41531828 | A/G | EP300 | missense | NM_001429.3 | c.1540A>G; p.Met514Val | VAL | N | ND | 0.00006589 | ND | ht | SM001_088 |
| 22:41574203 | C/T | EP300 | missense | NM_001429.3 | c.6488C>T; p.Pro2163Leu | VAL | N | ND | ND | ND | ht | MJ2003S |
| 22:44602262 | C/T | PARVG | stop_gained | NM_022141.6 | c.952C>T; p.Gln318Ter | VAL | N | rs73428406 | **0.001235 / AF 0.01357** | 0.006 | ht | MJ2013S |
| 22:45204262 | C/- | ARHGAP8 | frameshift | NM_001017526.1 | c.243delC; p.Arg82fs | VAL | N | ND | ND | ND | ht | MJ2004S |
| 3:119449131 | G/T | MAATS1 | stop_gained | NM_033364.3 | c.925G>T; p.Glu309Ter | VAL | N | ND | ND | ND | ht | MJ2013S |
| 3:12632426 | -/T | RAF1 | frameshift | NM_002880.3 | c.1241dupA; p.Asp415fs | VAL | N | ND | ND | ND | ht | MJ2016S |
| 3:137843333 | G/A | A4GNT | stop_gained | NM_016161.2 | c.796C>T; p.Arg266Ter | VAL | N | rs113881039 | 0.002389 | 0.001 | ht | MJ2015S |
| 3:14206341 | G/C | XPC | missense | NM_004628.4 | c.872C>G; p.Ser291Cys | VAL | N | rs184879571 | **0.002813 / EU 0.02986** | 0.001 | ht | MJ2015S |
| 3:188327445 | G/A | LPP | missense | NM_005578.3 | c.926G>A; p.Gly309Asp | VAL | N | rs146859876 | 0.001631 | 0.003 | ht | MJ1011S |
| 3:197241169 | C/- | BDH1 | frameshift | NM_004051.4 | c.528delG; p.Lys177fs | VAL | N | ND | ND | ND | ht | MJ2037S |
| 3:37089130 | AA/GC | MLH1 | missense | NM_000249.3 | c.1852_1853delinsGC; p.Lys618Ala | VAL | N | rs35502531 | 0.003418 | ND | ht | SM001_068 |
| 3:40085726 | A/- | MYRIP | frameshift | NM_015460.2 | c.296delA; p.Lys100fs | NE | NE | ND | ND | 0.004 | ht | MJ2001S/  SM001_088 |
| 3:48716158 | G/A | NCKIPSD | missense | NM_016453.3 | c.1804C>T; p.Arg602Cys | NE | NE | ND | 0.000008 | 0.001 | ht | MJ2007S/  MJ2012S |
| 3:49936697 | C/G | MST1R | splice_acceptor | NM_002447.2 | c.1231-1G>C; p.spl? | NE | NE | ND | 0.00008237 | ND | ht | SM001_068 |
| 3:52383038 | C/A | DNAH1 | stop_gained | NM_015512.4 | c.2241C>A; p.Tyr747Ter | VAL | N | ND | 0.00002473 | ND | ht | MJ2001S |
| 3:52833787 | A/T | ITIH3 | stop_gained | NM_002217.3 | c.925A>T; p.Arg309Ter | VAL | Y | ND | ND | ND | ht | MJ2037S |
| 4:106158350 | A/C | TET2 | missense | NM_001127208.2 | c.3251A>C; p.Gln1084Pro | VAL | N | rs75056899 | 0.002636 | 0.002 | ht | MJ2004S |
| 4:187455355 | G/A | MTNR1A | stop_gained | NM_005958.3 | c.541C>T; p.Gln181Ter | VAL | N | ND | ND | ND | ht | MJ2015S |
| 4:55963918 | C/T | KDR | missense | NM_002253.2 | c.2525G>A; p.Arg842His | VAL | N | rs149901681 | 0.0001236 | 0.001 | ht | MJ2037S |
| 4:55964925 | G/A | KDR | missense | NM_002253.2 | c.2312C>T; p.Thr771Met | VAL | N | rs149745504 | 0.001096 | 0.005 | ht | MJ2037S |
| 4:69342110 | TTAA/- | TMPRSS11E | frameshift | NM_014058.3 | c.661_664delTTAA; p.Ile222fs | VAL | N | ND | 0.0001647 | ND | ht | SM001_021 |
| 5:112163681 | C/T | APC | missense | NM_000038.5 | c.1604C>T; p.Ser535Phe | VAL | N | rs75870842 | 0.0002389 | ND | ht | MJ1011S |
| 5:114916260 | G/A | TICAM2 | stop_gained | NM_021649.6 | c.694C>T; p.Gln232Ter | VAL | N | ND | 0.0001153 | 0.001 | ht | SM001_040 |
| 5:132015535 | C/T | IL4 | stop_gained | NM_000589.3 | c.313C>T; p.Arg105Ter | VAL | N | ND | ND | ND | ht | MJ2037S |
| 5:13845086 | G/A | DNAH5 | stop_gained | NM_001369.2 | c.5131C>T; p.Arg1711Ter | VAL | Y | ND | 0.000008 | 0.001 | ht | MJ2013S |
| 5:141021096 | G/- | FCHSD1 | frameshift | NM_033449.2 | c.2042delC; p.Pro681fs | NE | NE | ND | ND | ND | ht | SM001_040 |
| 5:142292845 | G/A | ARHGAP26 | missense | NM_015071.4 | c.1015G>A; p.Glu339Lys | NE | NE | ND | 0.00002471 | 0.001 | ht | SM001_068 |
| 5:149514363 | A/G | PDGFRB | missense | NM_002609.3 | c.581T>C; p.Ile194Thr | VAL | N | rs2229560 | 0.001227 | 0.002 | ht | MJ2007S/  MJ2012S |
| 5:176308480 | A/- | HK3 | frameshift | NM_002115.2 | c.2450delT; p.Leu817fs | NE | NE | ND | ND | 0.001 | ht | SM001_040 |
| 5:34911884 | C/T | RAD1 | missense | NM_002853.3 | c.341G>A; p.Gly114Asp | NE | NE | rs2308957 | **0.005617 / FN 0.0124** | 0.003 | ht | SM001_088 |
| 5:35867562 | A/C | IL7R | missense | NM_002185.3 | c.376A>C; p.Ile126Leu | VAL | N | ND | 0.00009061 | ND | ht | MJ2003S |
| 5:40716359 | G/C | TTC33 | stop_gained | NM_012382.2 | c.677C>G; p.Ser226Ter | VAL | N | rs140209301 | 0.001606 | ND | ht | SM001_021 |
| 5:54423155 | G/A | CDC20B | missense | NM_001170402.1 | c.919C>T; p.Arg307Trp | NE | NE | rs145168670 | 0.004258 | 0.008 | ht | MJ1007S/  SM001_068 |
| 5:80109406 | G/A | MSH3 | missense | NM_002439.4 | c.2659G>A; p.Asp887Asn | NE | NE | ND | 0.00002471 | ND | ht | SM001_088 |
| 5:82400773 | C/G | XRCC4 | missense | NM_022406.2 | c.35C>G; p.Ser12Cys | NE | NE | rs28383138 | **0.002331 / AF 0.02543** | 0.002 | ht | SM001_088 |
| 6:10557208 | C/G | GCNT2 | stop_gained | NM_001491.2 | c.552C>G; p.Tyr184Ter | NE | NE | ND | 0.00005765 | ND | ht | MJ2015S |
| 6:110778048 | C/- | SLC22A16 | frameshift | NM_033125.3 | c.226delG; p.Ala76fs | VAL | N | ND | ND | ND | ht | MJ2007S/ MJ2012S |
| 6:111621304 | T/A | REV3L | missense | NM_002912.3 | c.9308A>T; p.Asn3103Ile | NE | NE | ND | ND | ND | ht | MJ2003S |
| 6:111697900 | A/G | REV3L | missense | NM_002912.3 | c.1658T>C; p.Leu553Pro | NE | NE | rs140709825 | 0.0000659 | 0.001 | ht | SM001_040 |
| **6:117622137** | **C/T** | **ROS1#** | **missense** | **NM_002944.2** | **c.6733G>A; p.Gly2245Ser** | **VAL** | **N** | **rs142264513** | **0.000766** | **0.002** | **ht** | **MJ2007S/**  **MJ2012S** |
| 6:117715381 | A/G | ROS1 | missense | NM_002944.2 | c.1108T>C; p.Ser370Pro | VAL | N | rs56274823 | 0.001639 | ND | ht | MJ2007S/  MJ2012S |
| 6:119136178 | G/A | MCM9 | stop_gained | NM_017696.2 | c.3241C>T; p.Arg1081Ter | VAL | N | ND | 0.00004689 | ND | ht | MJ2004S |
| 6:144508563 | G/A | STX11 | missense | NM_003764.3 | c.799G>A; p.Val267Met | NE | NE | rs45574234 | 0.005495 | 0.006 | ht | MJ2013S |
| 6:168351978 | A/T | MLLT4 | missense | NM_001207008.1 | c.3872A>T; p.Gln1291Leu | NE | NE | rs144979147 | **0.001153 / AF 0.01279** | 0.006 | ht | SM001_040 |
| 6:17649494 | G/A | NUP153 | missense | NM_001278209.1 | c.1526C>T; p.Pro509Leu | NE | NE | rs61756067 | **0.006457 / FN 0.02162** | 0.004 | ht | MJ2015S |
| 6:36068041 | T/- | MAPK14 | frameshift | NM_001315.2 | c.759delT; p.His253fs | NE | NE | ND | ND | ND | hm | SM001_040 |
| 7:105189122 | T/A | RINT1 | missense | NM_021930.4 | c.961T>A; p.Phe321Ile | NE | NE | ND | ND | ND | ht | MJ2003S |
| 7:107763575 | C/T | LAMB4 | splice_donor | NM_007356.2 | c.34+1G>A; p.spl? | NE | NE | rs7788865 | **0.001474 / AF 0.0166** | 0.003 | ht | MJ2004S |
| 7:116380055 | G/A | MET | missense | NM_001127500.1 | c.1444G>A; p.Asp482Asn | VAL | N | ND | ND | ND | ht | SM001_040 |
| 7:116380062 | A/G | MET | missense | NM_001127500.1 | c.1451A>G; p.His484Arg | VAL | N | ND | 0.00004966 | ND | ht | MJ2007S/  MJ2012S |
| 7:127239434 | G/C | FSCN3 | splice_acceptor | NM_020369.2 | c.1121-1G>C; p.spl? | NE | NE | rs147048987 | 0.00028 | ND | ht | SM001_068 |
| 7:23347569 | -/GTAA | MALSU1 | frameshift | NM_138446.1 | c.517_517+1insGTAA; p.Ser174fs | NE | NE | ND | 0.00007413 | ND | ht | SM001_068 |
| 7:25175985 | TGGTC/- | C7orf31 | frameshift | NM_138811.3 | c.1375_1379delGACCA; p.Asp459fs | VAL | N | ND | 0.00008237 | 0.002 | ht | SM001_021 |
| 7:45148837 | G/- | TBRG4 | frameshift | NM_001261834.1 | c.33delC; p.Met12fs | VAL | N | rs36007488 | **0.007497 / EU 0.01127** | 0.007 | ht | SM001_088 |
| 7:64439514 | -/G | ZNF117 | frameshift | NM_015852.3 | c.435dupC; p.Phe146fs | VAL | N | ND | 0.0002475 | ND | ht | MJ1011S |
| 7:91726522 | C/T | AKAP9 | missense | NM_005751.4 | c.10249C>T; p.Arg3417Cys | VAL | N | rs146495719 | 0.00007413 | 0.001 | ht | MJ2004S |
| 7:95024007 | G/A | PON3 | stop_gained | NM_000940.2 | c.94C>T; p.Arg32Ter | VAL | N | rs147006695 | 0.001417 | ND | ht | MJ2016S |
| 7:97921997 | -/AC | BRI3 | frameshift | NM_001159491.1 | c.291_292insAC; p.99fs | NE | NE | ND | 0.0003463 | 0.002 | ht | SM001_068 |
| 8:100899792 | C/T | COX6C | missense | NM_004374.3 | c.169G>A; p.Asp57Asn | NE | NE | ND | 0.00003295 | ND | ht | SM001_088 |
| 8:104240298 | C/T | BAALC | stop_gained | NM_024812.2 | c.409C>T; p.Arg137Ter | VAL | N | rs144334771 | 0.00005766 | ND | ht | MJ2037S |
| 8:10465490 | C/A | RP1L1 | stop_gained | NM_178857.5 | c.6118G>T; p.Glu2040Ter | VAL | N | rs201774530 | 0.00007432 | ND | ht | MJ2001S |
| 8:17815082 | T/G | PCM1 | missense | NM_006197.3 | c.1838T>G; p.Ile613Ser | NE | NE | rs181777656 | **0.002912 / OT 0.01174** | 0.002 | ht | MJ2007S/  MJ2012S |
| 8:22972272 | T/- | TNFRSF10C | frameshift | NM_003841.3 | c.269delT; p.Cys91fs | VAL | N | ND | 0.003171 | 0.003 | ht | SM001_040 |
| 8:30704364 | C/- | TEX15 | frameshift | NM_031271.3 | c.2170delG; p.Ala724fs | NVal | N | ND | ND | ND | ht | SM001_040 |
| 8:3072107 | G/- | CSMD1 | frameshift | NM_033225.5 | c.4779delC; p.Tyr1594fs | NVal | N | ND | ND | ND | ht | SM001_040 |
| 8:30922511 | A/G | WRN | missense | NM_000553.4 | c.436A>G; p.Lys146Glu | VAL | N | ND | 0.00002471 | ND | ht | SM001_040 |
| 8:52370127 | A/- | PXDNL | frameshift | NM_144651.4 | c.913delT; p.Cys305fs | NE | NE | rs146964162 | **0.002747 / AF 0.03204** | 0.007 | ht | SM001_021 |
| 8:71068718 | C/T | NCOA2 | missense | NM_006540.2 | c.1882G>A; p.Gly628Arg | VAL | N | rs201963563 | 0.000752 | 0.003 | ht | MJ1011S |
| 8:80677967 | C/T | HEY1 | missense | NM_001040708.1 | c.383G>A; p.Ser128Asn | NE | NE | ND | 0.00002471 | ND | ht | SM001_021 |
| 9:130166017 | C/T | SLC2A8 | stop_gained | NM_014580.4 | c.802C>T; p.Gln268Ter | VAL | N | rs41276194 | 0.0009476 | 0.004 | ht | MJ2014S |
| 9:139635307 | G/C | LCN10 | stop_gained | NM_001001712.2 | c.456C>G; p.Tyr152Ter | VAL | N | rs146003132 | 0.00004942 | ND | ht | MJ2037S |
| 9:139754430 | C/- | MAMDC4 | frameshift | NM_206920.2 | c.3286delC; p.Arg1096fs | VAL | N | ND | 0.00003305 | 0.001 | ht | SM001_040 |
| 9:140007688 | G/A | DPP7 | stop_gained | NM_013379.2 | c.673C>T; p.Arg225Ter | VAL | N | rs148933573 | 0.0001318 | ND | ht | SM001_088 |
| 9:18928286 | G/A | FAM154A | stop_gained | NM_153707.2 | c.1189C>T; p.Arg397Ter | VAL | Y | rs116103698 | 0.0006 | 0.004 | ht | SM001_088 |
| 9:34256126 | AC/- | KIF24 | frameshift | NM_194313.2 | c.3478_3479delGT; p.Val1160fs | VAL | N | ND | 0.00003295 | ND | ht | SM001_068 |
| 9:35616596 | C/T | CD72 | splice_donor | NM_001782.2 | c.352+1G>A; p.spl? | NE | NE | ND | ND | ND | ht | SM001_068 |
| 9:6255967 | G/C | IL33 | splice_acceptor | NM_033439.3 | c.613-1G>C; p.spl? | NE | NE | rs146597587 | 0.002 | 0.001 | ht | MJ2007S/  MJ2012S |
| 9:8501026 | G/A | PTPRD | missense | NM_002839.3 | c.1856C>T; p.Thr619Ile | VAL | N | ND | ND | 0.001 | ht | MJ2007S/  MJ2012S |
| 9:97535299 | T/- | C9orf3 | frameshift | NM_001193329.1 | c.813delT; p.Val272fs | VAL | N | ND | ND | ND | ht | MJ2001S |
| X:24861673 | T/C | POLA1 | missense | NM_016937.3 | c.3908T>C; p.Met1303Thr | VAL | N | ND | ND | ND | ht | MJ2007S/  MJ2012S |
| X:48118043 | A/G | SSX1 | missense | NM_005635.3 | c.257A>G; p.Asn86Ser | NVal | N | ND | ND | ND | ht | MJ2007S/ MJ2012S |
| X:76938264 | C/G | ATRX | missense | NM_000489.3 | c.2484G>C; p.Met828Ile | VAL | N | ND | 0.00005766 | ND | ht | MJ2015S |

Chr: chromosome; Pos: position; Ref: reference allele; Alt: alternate allele; VAL: validated variant; NE: not evaluated; NVal: not validated; CTLs: controls; N: no; Y: yes; ND: not described; MAF: minor allele frequency; ht: heterozygous; hm: homozygous. EU: European (Non-Finnish); LA: Latino; AF: African; FN: European (Finnish); OT: Others

# Variants predicted to have a potential effect on splicing according to dbscSNV (ADA and/or RF score >0.6).

**Supplementary table 3: Clinical features of the sixteen HBC patients selected for WES**

| **Patient** | **Age of onset** | **Neoplasia** | **Histologic type** | **ER/PR/HER2 status** | **Family history** |
| --- | --- | --- | --- | --- | --- |
| SUBJ1007 | 25 | BC | IDC | TNBC | Paternal great-aunt CRC 26y |
| SUBJ1011 | 33 | BC | ILC | ER+/PR+/HER2- | Positive (no further information available) |
| MJ2001 | 28 | BC | IDC | ER+/PR+/HER2+ | Mother BC 39y; maternal grandmother BC 53y; paternal uncle GC 66y |
| MJ2003 | 26 | BC | IDC | TNBC | Father PC 51y |
| MJ2004 | 34 | BC | IDC | ER+/PR+/HER2- | Mother BC 51y; maternal aunt CNS 46y; maternal aunt OV ?y |
| MJ2007* | 29 | BC | IDC | ER+/PR+/HER2- | Sister BC 29y; father LC 59y; two paternal uncles LC 32y and 45y; paternal aunt OV 45y; paternal grandmother PaC 47y; paternal grandfather LC 60y; maternal grandfather CRC 62y; maternal great-aunt BC 42y; maternal great grandmother UC 30y |
| MJ2012* | 29 | BC | IDC | TNBC | Sister of patient MJ2007 |
| MJ2013 | 29 | BC | IDC | ER+/PR+/HER2- | Paternal grandmother BC 47a; three paternal great-aunts BC 67y, 50y and 35y; paternal great-uncle BC 60y; 6 four or five degree paternal relatives with BC <50y |
| MJ2014 | 35 | BC | MED | TNBC | Maternal uncle CRC 50y |
| MJ2015 | 34 | BC | IDC | ER+/PR+/HER2+ | Paternal aunt BC 50y |
| MJ2016 | 29 | BC | IDC | ER+/PR+/HER2+ | Paternal great aunt BC 50y, one paternal fourth degree relative with OS 30y |
| MJ2037 | 27 | BC | IDC | TNBC | Paternal grandmother BC 55y |
| SM001.021 | 21 | OV (bilateral) | - | - | Maternal aunt OV and BC 53y/62y; paternal uncle CNS ?y, paternal grandmother BC 75y |
| SM001.040 | 51 | BC | NA | NA | Mother BC 35y, 1 sister BC 35y, 1 sister BC 52y, 1 sister TC 45y, 1 sister OV ?y |
| SM001.049 | 29/34 | Melanoma/BC | MUC | ER-/PR+/HER2- | Mother BC 48y; sister BC 36y; maternal aunt BC ?y |
| SM001.068 | 37/44 | BC/ OV | NA | NA | Negative family history |
| SM001.088 | 35 | BC | IDC | ER+/PR+/HER2(NA) | Maternal aunt BC 62y; two maternal third degree relatives with GC 60 and 28y; one maternal third degree relative with CRC ?y; three maternal third degree relatives BC ?y |

*Sisters; IDC: Invasive ductal carcinoma; ILC: Invasive lobular carcinoma; MED: medullary breast cancer; MUC: mucinous breast cancer; TNBC: triple negative breast cancer; ER: estrogen receptor; PR: progesterone receptor; neg: negative; pos: positive; NA: not available; BC: breast cancer, OV: ovary cancer; TC: thyroid cancer; CNS: central nervous system cancer; GC: gastric cancer; PC: prostate cancer; KC: kidney cancer; UC: uterine cancer; LK: leukemia; PaC: pancreatic cancer; OS: osteosarcoma; LC: liver cancer

**Supplementary table 4: WES statistics**

| **Sample** | **Platform** | **Reads on targets** | **% Targets covered** | **% Targets coverage >10X** | **% Targets coverage >20X** |
| --- | --- | --- | --- | --- | --- |
| **Hereditary breast cancer cases** | | | | | |
| MJ1007S | SOLID | 16,854,403 | 80.89 | 56.34 | 38.86 |
| MJ1011S | SOLID | 41,569,796 | 90.83 | 78.17 | 69.23 |
| MJ2001S | SOLID | 21,478,980 | 79.34 | 44.02 | 26.76 |
|  | Proton | 55,683,649 | 98.10 | 88.34 | 76.43 |
| MJ2003S | SOLID | 85,493,240 | 94.62 | 84.17 | 77.33 |
| MJ2004S | SOLID | 68,991,869 | 89.81 | 74.93 | 64.77 |
| MJ2007S* | SOLID | 29,981,900 | 88.37 | 70.52 | 57.88 |
| MJ2012S* | SOLID | 32,826,544 | 90.40 | 75.43 | 62.73 |
| MJ2013S | SOLID | 44,918,683 | 90.19 | 76.85 | 67.51 |
| MJ2014S | SOLID | 64,255,635 | 95.09 | 85.96 | 79.07 |
| MJ2015S | SOLID | 26,430,667 | 88.54 | 70.52 | 56.56 |
| MJ2016S | SOLID | 28,316,458 | 88.82 | 71.52 | 58.76 |
| MJ2037S | SOLID | 158,497,176 | 92.41 | 84.49 | 80.03 |
| SM001.021 | SOLID | 18,888,071 | 81.91 | 56.19 | 38.40 |
|  | Proton | 50,647,640 | 98.46 | 94.59 | 91.71 |
| SM001.040 | Proton | 43,449,000 | 98.45 | 94.28 | 91.30 |
| SM001.049 | SOLID | 24,891,676 | 82.47 | 59.77 | 44.09 |
|  | Proton | 56,086,743 | 98.27 | 89.89 | 80.60 |
| SM001.068 | SOLID | 17,480,288 | 81.51 | 54.81 | 36.58 |
|  | Proton | 55,702,353 | 98.42 | 95.00 | 92.87 |
| SM001.088 | SOLID | 29,074,293 | 85.98 | 66.11 | 51.26 |
|  | Proton | 47,244,323 | 98.23 | 94.20 | 91.08 |
| **Mean** |  | **46,307,427** | **90.50** | **75.73** | **65.27** |
| ***BRCA1*-Mutated patients** | | | | | |
| MJ1014S | SOLID | 54,055,491 | 91.28 | 80.28 | 72.43 |
| MJ2021S | SOLID | 18,778,450 | 79.21 | 54.11 | 36.84 |
| MJ2026S | SOLID | 62,233,792 | 87.05 | 75.75 | 67.22 |
| MJ2034S | SOLID | 106,241,670 | 94.64 | 84.57 | 78.42 |
| MJ4010S | SOLID | 20,647,504 | 80.73 | 60.45 | 45.52 |
| **Mean** |  | **52,391,381** | **86.58** | **71.03** | **60.09** |

**A**


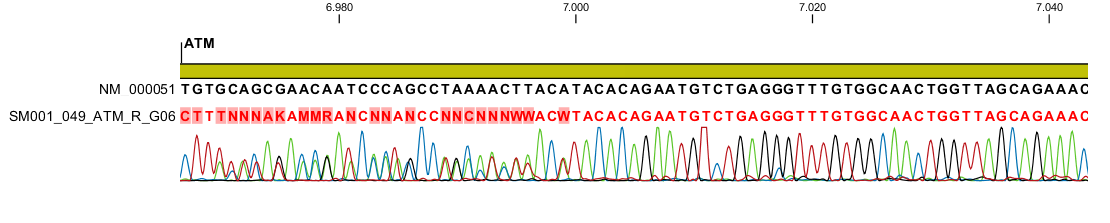


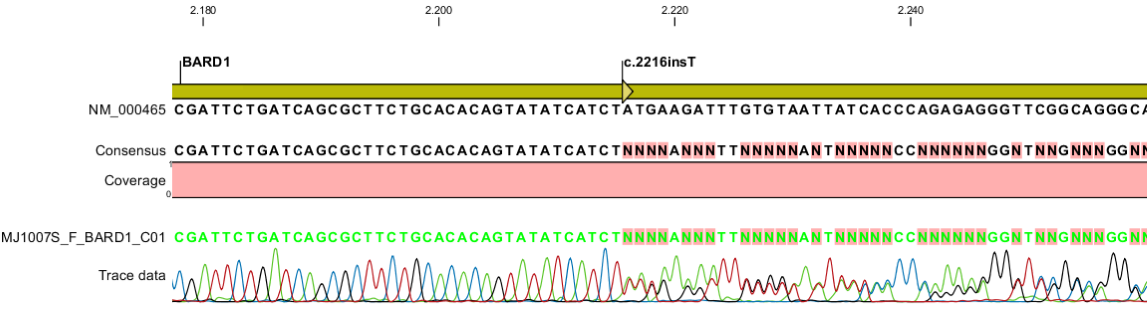


**B**

**Supplementary figure 1: *ATM* and *BARD1* validation by Sanger Sequencing.** Electropherogram of *ATM* c.7000_7003delTACA; p.(Tyr2334Glnfs*4) (NM_000051.3) pathogenic mutation in patient SM001.049. **B.** Electropherogram of *BARD1* c.2215dupT p.(Tyr739Leufs*2) (NM_000465.3) probably pathogenic mutation in patient SUBJ1007.
